# Progress on Catalyst Development for the Steam Reforming of Biomass and Waste Plastics Pyrolysis Volatiles: A Review

**DOI:** 10.1021/acs.energyfuels.1c01666

**Published:** 2021-08-04

**Authors:** Laura Santamaria, Gartzen Lopez, Enara Fernandez, Maria Cortazar, Aitor Arregi, Martin Olazar, Javier Bilbao

**Affiliations:** † Department of Chemical Engineering, University of the Basque Country UPV/EHU, P.O. Box 644, E48080 Bilbao, Spain; ‡ IKERBASQUE, Basque Foundation for Science, María Díaz de Haro 3, 48013 Bilbao, Spain

## Abstract

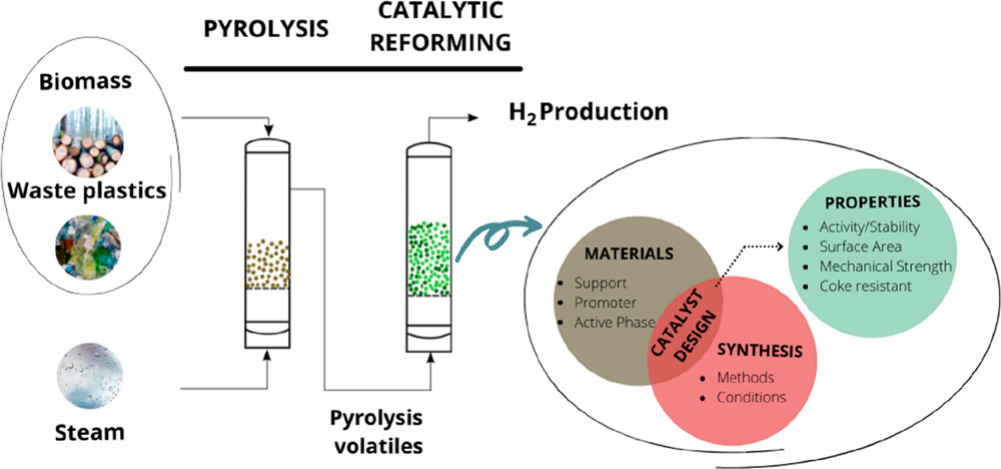

In recent decades, the production of H_2_ from biomass, waste plastics, and their mixtures has attracted increasing attention in the literature in order to overcome the environmental problems associated with global warming and CO_2_ emissions caused by conventional H_2_ production processes. In this regard, the strategy based on pyrolysis and in-line catalytic reforming allows for obtaining high H_2_ production from a wide variety of feedstocks. In addition, it provides several advantages compared to other thermochemical routes such as steam gasification, making it suitable for its further industrial implementation. This review analyzes the fundamental aspects involving the process of pyrolysis-reforming of biomass and waste plastics. However, the optimum design of transition metal based reforming catalysts is the bottleneck in the development of the process and final H_2_ production. Accordingly, this review focuses especially on the influence the catalytic materials (support, promoters, and active phase), synthesis methods, and pyrolysis-reforming conditions have on the process performance. The results reported in the literature for the steam reforming of the volatiles derived from biomass, plastic wastes, and biomass/plastics mixtures on different metal based catalysts have been compared and analyzed in terms of H_2_ production.

## 1 Introduction

The growing energy demand and the increasing awareness of the dependency on fossil fuels are promoting the use of alternative routes for the production of clean energy from sustainable raw materials and consumer society wastes. Currently, almost 80% of the global primary energy demand is supplied from crude oil, natural gas, and coal.^1^ Thus, the development of H_2_ technologies can help to alleviate the problems associated with global warming and climate change, promote sustainable development, and help to reduce CO_2_ emissions.

**Figure 1:**
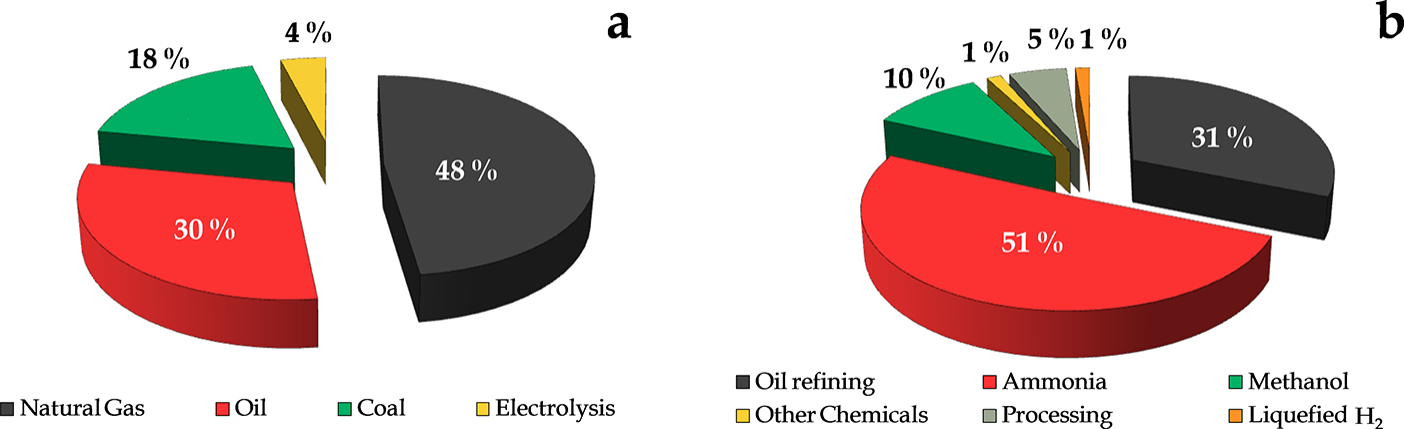
Global current sources of H_2_ production (a), and H_2_ consumption sectors (b).

The current global H_2_ production is about 8 EJ/year, with around 96% being produced from fossil fuels. Thus, the actual sources whereby H_2_ is produced consist of natural gas (48%), oil (30%), coal (18%), and water electrolysis (4%) (see Figure 1a). This distribution shows that the current H_2_ generation is associated with the consumption of fossil fuels and the emission of CO_2_ in their production processes (mostly steam reforming processes). In addition, its consumption is largely carried out in the petrochemical industry for the generation of automotive fuels, which in turn are CO_2_ generators.

Almost all the H_2_ produced is used for the production of existing feedstocks, i.e., in refineries or in the production of ammonia for urea or other fertilizers or chemicals, such as methanol. Thus, the global H_2_ consumption is distributed as follows: (i) ammonia production for urea and other fertilizers (51%); (ii) refining, hydrocracking, hydrotreating (e.g., fuel desulfurization), biorefinery (31%); (iii) synthesis of methanol and derivatives (10%); (iv) processing, (e.g., rocket fuel, automotive fuel or semiconductor industry (5%); (v) production of other chemicals, (e.g., polymers, polyurethanes, fatty acids (1%); and (vi) liquified H_2_ for steel heat treating, metal welding and forming, blanketing gas, and glass production (1%). These results are summarized in Figure 1b.

The future of the H_2_ market is driven by the need to reduce CO_2_ emissions, which requires its production from alternative raw materials and renewable sources. Thus, in order to achieve the global target signed in the Paris Agreement in 2015, in which the aim was to limit the global temperature rise in this century to 2 °C, dramatic changes should be done in order to decrease CO_2_ emissions by 60% until 2050.^2,3^ In this scenario, the short-term implementation of H_2_ production processes from biomass and waste is gaining growing attention. Consequently, the development of technologies for obtaining H_2_ from biomass and waste, especially waste plastics, has deserved remarkable attention in the recent literature.^4−10^ Among them, thermochemical routes have proven a great potential and efficiency. These processes can be grouped into direct and indirect routes. Thus, the direct routes are classified as follows: (i) pyrolysis (catalytic pyrolysis and high temperature pyrolysis), (ii) steam gasification, (iii) supercritical water gasification, and (iv) the steam reforming of pyrolysis volatiles. The indirect routes are those involving intermediate steps to obtain liquid products (bio-oil or plastics pyrolysis oil) for their subsequent reforming (steam reforming and gasification).

Steam gasification has been widely studied in the literature for the valorization of biomass and waste plastics, since it can generate H_2_-rich syngas^9,11,12^ and the gaseous products can be directly used as fuel or intermediate products in the large scale chemical and fuel production.^13,14^ The main challenge of gasification processes involves the quality of the syngas and its tar content and, in fact, remarkable research efforts have been made to improve tar elimination processes.^15−17^ Thus, it is well-established that an optimized gasifier and a highly active catalyst can efficiently contribute to biomass tar elimination.^18^ Under suitable operating conditions and using appropriate catalysts, biomass steam gasification allows obtaining H_2_ productions in the 5–7 wt % range.^19−22^ The higher carbon and hydrogen content of waste plastics increases H_2_ production to values above 10 wt % when they are valorized by steam gasification.^23−25^

An alternative strategy to direct gasification proposed for syngas production is the one based on the gasification of pyrolysis oil, especially the one derived from biomass pyrolysis. This process involves the advantage of reducing the expensive transportation costs of the biomass and waste.^26^ Although the differentiation in the literature between bio-oil gasification and reforming is sometimes unclear and rather confusing, it has herein considered that bio-oil gasification is the process that requires higher temperatures (around 800–1400 °C) than reforming reactions and is carried out without catalysts or in the presence of primary mineral catalysts.^8^ Thus, the composition of the syngas obtained in the bio-oil gasification is similar to the one obtained in the biomass gasification, with higher H_2_ yields being attained in the gas product from bio-oil reforming. The production of H_2_ from bio-oil gasification is greatly influenced by the type of bio-oil used, reactor configuration, and operating conditions. Accordingly, H_2_ production values from 1.4 to 12.6 wt % have been reported in the literature.^27−30^

Bio-oil steam reforming is an indirect thermochemical route for H_2_ production from biomass.^31,32^ It should be noted that this strategy has been scarcely studied as a route for the upgrading of waste plastics. The bio-oil or liquid product from biomass pyrolysis has a higher energy density compared to biomass, leading to lower transportation costs and therefore allowing bio-oil valorization in centralized large scale catalytic conversion units. In spite of the operational problems associated with bio-oil handling and fast deactivation rate of the reforming catalyst, high H_2_ productions have been reported. Thus, values above 12 wt % by mass unit of the organic bio-oil have been obtained.^33−36^ The selection of suitable catalysts for oxygenate reforming has been extensively studied in the literature by using model compounds, the bio-oil aqueous phase, and raw bio-oil.^37,38^ Thus, although a wide range of base transition metals, such as Ni, Co, and Fe, and noble metals, such as Rh, Pt, Ir, and Ru, have been studied, Ni based catalysts are the most used ones because they strike a suitable balance between activity and cost.^39,40^ In addition, different supports and their modification with promoters have also been widely analyzed.^12,41,42^

An alternative and direct thermochemical conversion route for H_2_ production from biomass and waste is the strategy based on pyrolysis and in-line reforming of the volatiles, which has several advantages in comparison with the aforementioned routes. Thus, the integration of both reactors in the same unit allows selecting the optimum conditions in the pyrolysis and in-line reforming steps.^43^ Therefore, operation at lower temperatures than those in the gasification process and the use of highly active reforming catalysts lead to the avoidance of tar formation, reduce material costs, and prevent catalyst deactivation by sintering.^44−46^ Moreover, the direct contact of the reforming catalyst with the biomass and its impurities is avoided, given that they are retained in the pyrolysis reactor.^47^ In addition, this process has shown a remarkable capacity for H_2_ production. Concerning biomass conversion, values of around 10 wt % were obtained operating under optimum conditions and catalysts.^40,48−53^ The volatiles derived from biomass pyrolysis can be classified into two fractions: (i) bio-oil or liquid fraction (the condensable fraction at the outlet of the pyrolyzer), which is formed by a complex mixture of oxygenated compounds (phenols, ketones, saccharides, furans, acids, and alcohols) and water, and (ii) the non-condensable gaseous stream, which is mainly made up of CO and CO_2_ and, to a minor extent, by light hydrocarbons and H_2_. In the case of waste plastics, a wide range of conversions was reported depending on the polymer nature. Thus, polyolefins and polystyrene allow obtaining H_2_ production values higher than 30 wt %,^43,46,54,55^ whereas other polymers with lower carbon content, such as polyethylene terephthalate, lead to productions below 20 wt %.^56^ The volatile stream derived from polyolefins (PE and PP) pyrolysis under mild conditions was mainly formed by waxes (C_21_+) followed by hydrocarbons in the diesel fraction (C_12_–C_20_), with the yields of light oil (C_5_–C_11_) and gases being rather low. The products obtained in the pyrolysis of polystyrene (PS) are made up of aromatic hydrocarbons (styrene recovery being higher than 70%) and a small yield of light gaseous compounds. It is to note that no solid residue is formed in the pyrolysis of polyolefins and polystyrene, and therefore all the products that make up the volatile stream are fed into the reforming step. Conversely, a solid residue is obtained in the pyrolysis of PET, with the volatile stream being mainly composed of non-condensable gases (CO and CO_2_), oil (acetaldehyde), and a mixture of oxygenated compounds, which are solid at room temperature (mostly benzoic acid).

Therefore, H_2_ production by pyrolysis-reforming is highly dependent on the raw material. Furthermore, the volatile composition to be reformed affects the reaction mechanism as well as the catalyst deactivation rate.^57^

However, the development of the combined process of pyrolysis and in-line reforming is conditioned by the efficiency of the reforming catalyst. Thus, highly selective and active catalysts are required to enhance H_2_ production and ensure full conversion of pyrolysis volatiles and therefore avoid tar formation. Moreover, the complex nature of the volatile stream derived from the pyrolysis of biomass and waste plastics causes a fast deactivation rate.^58^ Thus, a remarkable research effort has been made in recent years to develop new catalysts and overcome the mentioned challenges. Accordingly, a wide variety of catalysts based on different supports, promoters, and active phases have been proposed in the literature. This review analyzes the development and application of these catalysts for the reforming of biomass and waste plastics. Moreover, a general overview on the combined process of pyrolysis and in-line reforming has also been included. The role played by pyrolysis and reforming conditions on process performance was briefly discussed, and technological aspects were also considered.

## 2 Pyrolysis and In-line Steam Reforming

**Figure 2:**
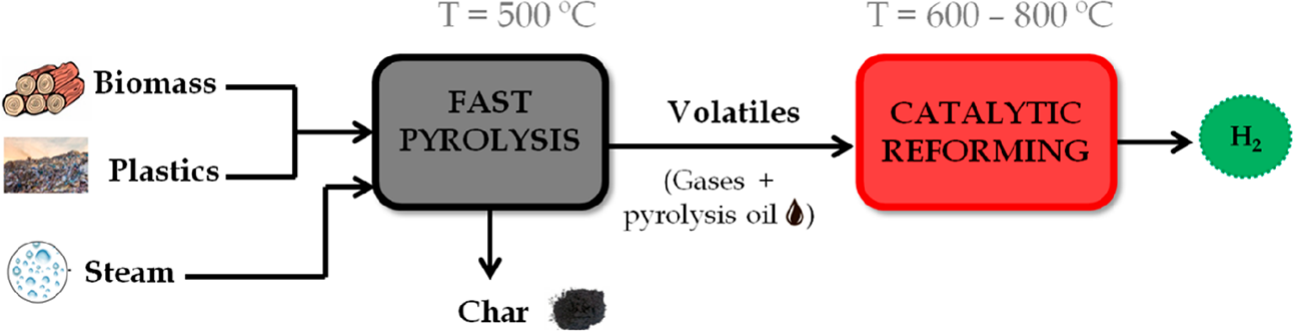
Scheme of pyrolysis-reforming of biomass/plastics.

The pyrolysis-reforming strategy pursues H_2_ production by combining two reaction steps in a single process (see Figure 2). The aim of the pyrolysis step is to convert the solid feedstock into a volatile stream suitable for further transformation into a H_2_ rich gas in the subsequent catalytic reforming step. The composition and yields of the pyrolysis volatiles (gas and pyrolysis oil) depend on the pyrolysis conditions and feedstock characteristics. Moreover, a solid product or char can also be formed in the pyrolysis step, which is a valuable byproduct that is not fed into the reforming reactor. The pyrolysis step is usually performed at around 500 °C, as this is the minimum temperature to ensure full devolatilization of biomass and waste plastics.

The step of catalytic steam reforming of the pyrolysis volatiles is commonly carried out between 600 and 800 °C. This temperature range is conditioned by, on the one hand, the low reaction rate below 600 °C and, on the other hand, the catalyst instability due to metal sintering when operation is carried out at high temperatures. Different supported metallic catalysts have been used in this process, with Ni based ones the most common due to their suitable activity and low price. Steam partial pressure and catalyst space time also play a significant role in the performance of the reforming step and must be carefully considered.

In the following sections, the most relevant aspects involving the steps of pyrolysis and reforming are briefly discussed. Moreover, the main reactor designs used in this process are also presented and analyzed.

### 2.1 Pyrolysis Step

Pyrolysis is a thermochemical conversion process performed under inert conditions. Although pyrolysis is usually performed under a steam atmosphere, the mild conditions used for pyrolysis in the combined process of pyrolysis-reforming minimize the impact of steam in the pyrolysis mechanism^44,50^ when it is used as an inert carrier gas. Pyrolysis processes are often divided into three groups depending on the heating rate and gas residence time, namely, fast, intermediate, and slow pyrolysis.^59,60^ Thus, fast pyrolysis is usually conducted at moderate temperatures under very high heating rates (10^3^–10^4^ °C s^–1^) and short residence times (below 1 s). These conditions minimize secondary reactions leading to the non-condensable gas fraction and enhance the yield of primary products accounting for the liquid fraction. However, slow pyrolysis is characterized by long residence times and slow heating rates. It is to note that these conditions favor the formation of gas and solid products at the expense of the liquid one. Finally, intermediate pyrolysis conditions are halfway between those of fast and slow pyrolysis. In the processes involving pyrolysis and reforming, the most common fast pyrolysis reactors are fluidized and spouted beds, and their use is associated with the operation under a continuous regime.^54,56,61,62^ However, fixed bed reactors are used under slow pyrolysis conditions (below 40 °C min^–1^) and they operate in a batch regime.^53,63,64^

Thermal pyrolysis of biomass and waste plastics leads to wide and complex product distributions. The features of the obtained products are briefly mentioned below due to their impact on the subsequent reforming step. Biomass pyrolysis leads to three fractions, namely, gas, bio-oil, and char, with their yields depending on the pyrolysis conditions and original biomass composition. Although biomass has a variable composition in terms of cellulose, hemicellulose, lignin, sugar, protein and ash contents, some general features apply in all cases. Thus, the gases are mainly made up of CO and CO_2_, with minor contents of light hydrocarbons and H_2_. Bio-oil is the main product obtained in biomass pyrolysis, with its yield reaching 75 wt % under suitable processing conditions.^59^ The bio-oil is a complex mixture of oxygenated compounds, with phenols, ketones, saccharides, furans, acids, or alcohols being worth mentioning. Moreover, a significant amount of water is also formed in the biomass thermal degradation. The char is a carbonaceous material obtained as a byproduct in the pyrolysis-reforming process, which is not treated in the reforming step. However, it has several applications, as are those related to sorbents, fertilizers, catalyst supports, and soil amender.^65,66^

The type of waste plastic fully conditions the pyrolysis mechanism and the obtained product distribution. Accordingly, the product distribution obtained in the pyrolysis of each type of plastic waste deserves an individual description. Polyolefins, such as polyethylene (PE) and polypropylene (PP), are the most used plastics, whose thermal degradation takes place via a random radical scission mechanism leading to a wide product distribution from light gaseous hydrocarbons to heavy waxes. Furthermore, under suitable operating conditions, the yield of solid residue from these plastics is negligible.^67,68^ It should be noted that, under mild pyrolysis conditions, waxes and hydrocarbons in the diesel range are the prevailing products, with the yields of light oil and gases being low.^69−71^ However, the thermal degradation of polystyrene (PS) is a highly selective process. In fact, styrene recovery is above 90%, with the remaining products being other aromatics.^72−74^ Polyethylene terephthalate (PET) is a commodity plastic whose valorization by pyrolysis is challenging. Thus, the product distribution is a complex mixture of oxygenates (gaseous, liquid, and solid at room temperature) and a variable yield of solid residue.^75,76^ The yield of gas is higher than 40% and consists of CO and CO_2_, whereas the main heavy compounds are oxygenates of aromatic nature, such as benzoic acid. The co-pyrolysis of waste plastics and biomass is attracting increasing attention in recent years due to the interaction between their pyrolysis products and therefore the synergistic effects observed in their joint treatment.^77^ In spite of these interactions, the composition of the products is basically that corresponding to the contribution of the individual raw materials pyrolyzed.

### 2.2 Catalytic Steam Reforming Step

In the steam reforming step, the volatile stream from the pyrolysis step reaches the catalytic bed and reacts with steam on the catalyst active sites to yield a H_2_ rich gaseous product. The most relevant reactions in the reforming reactor are summarized in Table 1. These reactions can be divided into two groups, namely, heterogeneous catalytic reactions and secondary reactions in the gas phase. The first group includes the main desired reactions, which are promoted by the use of metallic catalysts. Steam reforming reactions convert oxygenates (eq 3) and hydrocarbons (eqs 4 and 5) into H_2_ and CO. These reactions are highly endothermic, and they are therefore favored at high temperatures. Moreover, CO can be further oxidized to CO_2_ by the water gas shift (WGS) reaction (eq 6), forming additional H_2_. This reaction is exothermic and is therefore hindered at high temperatures.

**Table 1:** Main Reactions Involved in the Reforming of the Volatiles Derived from Biomass and Waste Plastics

Bio-oil cracking:	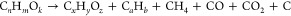 1
Hydrocarbons (HCs) cracking:	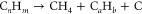 2
Bio-oil steam reforming:	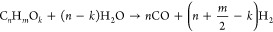 3
Methane steam reforming:	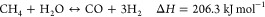 4
HCs steam reforming:	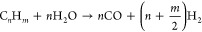 5
Water gas shift (WGS):	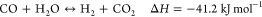 6
Interconversion:	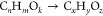 7

Secondary reactions include thermal cracking reactions of oxygenates (eq 1) and hydrocarbons (eq 2) and a wide variety of interconversion reactions (eq 7). These reactions are favored by severe operating conditions, i.e., high temperatures and long residence times. However, their impact is in general limited due to the competence with the much faster reactions promoted by the catalysts, i.e., steam reforming reactions. Thus, they only influence the final product composition when the process is performed under low conversion conditions (low space times or severely deactivated catalysts).

Apart from the conventional reforming under a steam atmosphere, the use of CO_2_ or dry reforming has been also proposed, especially when methane is used as the feedstock.^78^ Nevertheless, the low hydrogen content of biomass limits the interest of its valorization by dry reforming. However, the dry reforming of the volatiles derived from waste plastics leads to a significant H_2_ production.^79^ The oxidative steam reforming or oxygen co-feeding is a common strategy applied in catalytic steam reforming for the attenuation of the endothermicity of the process. Although the presence of oxygen in the reforming reactor contributes to reducing H_2_ production, it also has positive effects, such as the in situ combustion of the coke deposited on the catalysts and therefore, stability improvement.^80^

### 2.3 Pyrolysis-Reforming Reactor Configurations

**Figure 3:**
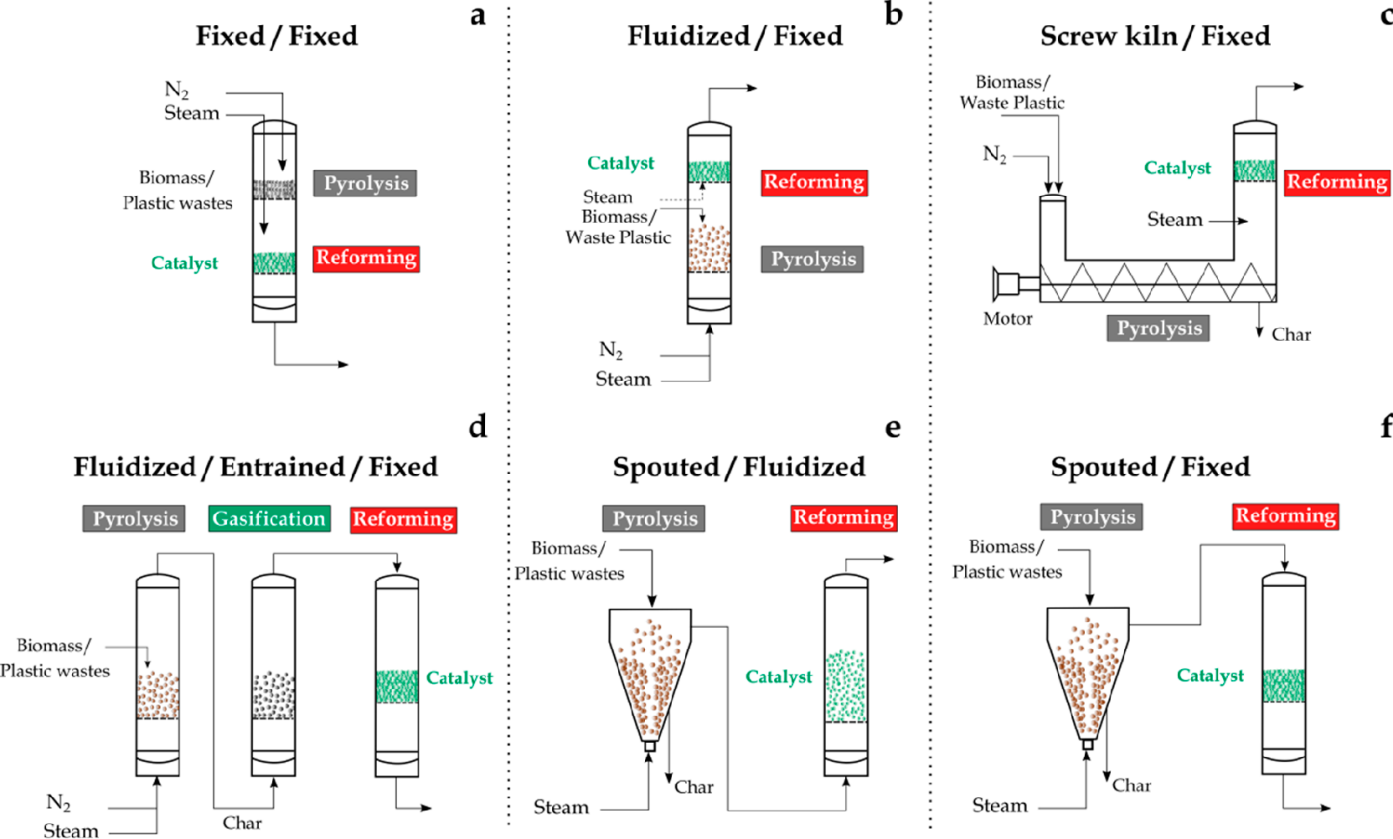
Reactor configurations for pyrolysis and in-line catalytic steam reforming of biomass and waste plastics: (a) fixed bed/fixed bed, (b) fluidized bed/fixed bed, (c) screw kiln/fixed bed, (d) fluidized bed/entrained flow/fixed bed, (e) spouted bed/fluidized bed, and (f) spouted bed/fixed bed. Adapted with permission from ref (8). Copyright 2018 Elsevier.

A wide variety of reactor configurations has been proposed in the literature for biomass and waste pyrolysis and in-line reforming. Figure 3 summarizes the main reactor designs used. However, the majority of the studies were carried out in batch laboratory units, and they are therefore of a preliminary nature.^9^ Nevertheless, continuous operation regime is highly relevant in the pyrolysis-reforming process. Thus, continuous pyrolysis leads to a volatile stream of constant composition once steady state conditions have been attained. This fact not only allows for the extrapolation of the results to the conditions of industrial reactors but also eases the evaluation of catalyst performance as a pyrolysis stream with homogeneous composition throughout the time treated. Moreover, steady state conditions also allow for a better control of the process conditions and catalyst stability with time on stream.

A combination of two fixed bed reactors for the pyrolysis and reforming steps has been widely used due to its simple design and operation as well as limited investment cost. The most common approach is the operation in a batch regime with the pyrolysis step being performed under slow pyrolysis conditions by using heating rates below 50 °C min^–1^.^47,81−85^ Thus, the pyrolysis volatiles formed throughout the heating process are transferred to the fixed bed reforming reactor, which operates under isothermal regime at the desired reforming temperature. The main shortcoming of this reactor configuration lies in its scaling up due to the poor heat transfer rate and complex control of operating conditions in the pyrolysis step. Besides, the use of a fixed bed reactor in the reforming step may involve operational problems related to bed blockage by coke deposition on the catalyst surface. Moreover, a two fixed bed configuration has also been used in the continuous regime for biomass^86−89^ and waste plastics^43,90^ valorization. It is to note that, in these studies, the continuous feed into the pyrolysis reactor leads to high heating rates. The use of fast pyrolysis reactors, such as fluidized and spouted beds, has also been proposed in the literature. These studies were carried out with a continuous biomass/plastic feed. The main advantages of these reactors are related to their suitable gas–solid contact features, high heat and mass transfer rates between phases and bed isothermicity. Fluidized bed reactors have limitations related to the feed and particle size in the bed, with defluidization being the main shortcoming, especially when plastic wastes are handled. The vigorous solid circulation movement in spouted beds allows handling coarse solids with irregular texture.^91,92^ The suitability of the conical spouted bed reactor has been proven in the pyrolysis of different residues, such as biomass, sewage sludge,^93^ tires,^94^ and plastics.^75^ In fact, a conical spouted bed reactor pilot plant (25 kg h^–1^) for biomass fast pyrolysis has been successfully operated.^95^ Thus, the pyrolysis in bubbling fluidized bed reactors was combined with the reforming of the volatiles in fixed^49^ and fluidized bed^54,96^ reactors. In the same line, the use of spouted beds in the pyrolysis step was also combined with fixed^97,98^ and fluidized bed^99−101^ reforming reactors. Moreover, Efika et al.^102^ used a screw kiln/fixed bed reactor configuration for the continuous biomass processing. Kuchonthara et al.^103^ studied biomass pyrolysis-reforming over a K_2_CO_3_/NiO/γ-Al_2_O_3_ catalyst in a batch reaction unit including a drop tube furnace and fixed bed for the pyrolysis and reforming steps, respectively.

It should be noted that the use of a fast pyrolysis reactor has practical advantages for the full scale operation in the pyrolysis-reforming process. Thus, it ensures an efficient conversion of biomass into volatiles with low yields of char^59,104^ and therefore increases the H_2_ production potential of the process. Moreover, these reactors are easy to operate and control in the continuous regime and allow for continuous char removal. In the same line, fluidized beds have advantages for the steam reforming process in relation to fixed beds. On the one hand, a better control of process conditions can be attained, which is essential in the case of temperature in this highly endothermic process. On the other hand, the pyrolysis-reforming process is greatly conditioned by the fast deactivation rate of the catalysts,^8,9,105^ and the use of a fluidized bed reactor allows implementing advanced catalyst regeneration strategies. Accordingly, a careful selection of the reactor design must be considered for full scale operation.

## 3 Reforming Catalysts

Although great effort has been devoted to the development of catalysts for the reforming of bio-oil model compounds, the studies conducted by feeding crude bio-oil are scarce. Similarly, few studies deal with the assessment of catalyst performance in the pyrolysis-reforming of plastic wastes. Therefore, knowledge on the performance of reforming catalysts under real process conditions, i.e., with a real pyrolysis volatile composition, is still limited. In fact, most of the studies have been carried out in batch-scale plants, and therefore further research effort is required in order to establish a suitable relationship between catalyst properties and their activity and stability in continuous large-scale plants.

Thus, this section deals with a brief introduction of heterogeneous catalysts, with special attention being paid to catalyst design (section 3.1) and the main causes and mechanisms of catalyst deactivation (section 3.2). Moreover, section 4 summarizes the aspects involving the catalysts used in the literature for biomass and plastic wastes pyrolysis and in-line reforming.

### 3.1 Catalyst Design

The design of a suitable catalyst for a specific process involves several challenges, as a wide range of features should be considered and optimized in order to achieve the desired performance. Accordingly, a large number of studies have been conducted on the reforming of bio-oil oxygenated compounds on commercial catalysts.^50,106,107^ However, the starting point for selecting possible materials and conditions for catalyst synthesis requires delving into the mechanisms of the process and the analysis and comparison of literature results.

#### 3.1.1 Catalyst Components

**Figure 4:**
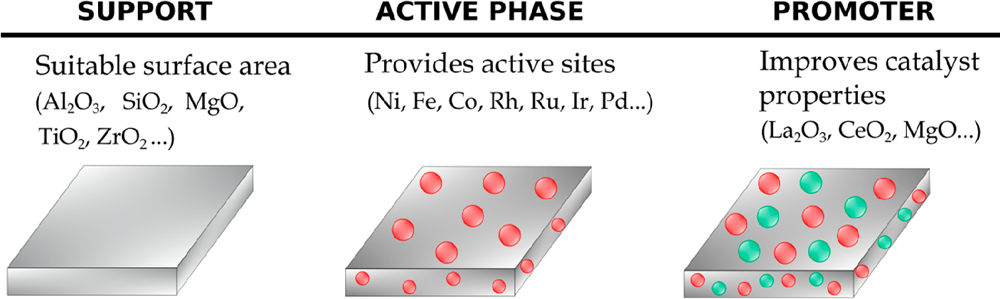
Catalyst components.

The main components of a typical heterogeneous catalyst are as follows (Figure 4): (i) active phase, metal that provides active sites for the chemical reaction; (ii) support or carrier, high specific surface area oxide or carbon on which the active phase is dispersed and stabilized; and (iii) promoter, additive that improves catalyst properties, i.e., activity, selectivity, and catalyst life.

Consequently, the selection of suitable catalytic materials is one of the most important factors for catalyst synthesis. Accordingly, given that active components are responsible for the main chemical reactions, the metal oxide selected as the active phase should promote reforming and WGS reactions in order to enhance H_2_ production in the reforming step. Ni-based catalysts have been widely used in the literature for CH_4_ and naphtha reforming^108,109^ as well as in the reforming of oxygenated compounds derived from biomass pyrolysis.^110−112^ Besides, other base transition metals, such as Co or Fe, and especially noble metals, such as Rh, Pt, Ir, and Ru, have been widely studied in the literature.^113−115^

Moreover, a suitable support must provide a high specific surface area, adequate pore distribution, mechanical strength, and good thermal stability. These two latter features are usually key factors in the steam reforming reactions.^116^ In addition, an ideal support should not have catalytic activity promoting secondary reactions leading to catalyst deactivation, as happens with Al_2_O_3_ support, which although is the most used support in reforming reactions, its acid properties promote coke deposition under reaction conditions and lead to catalyst deactivation.^117,118^ In this way, the support should facilitate the dispersion of the active phase and modulate catalyst activity.

The addition of the right promoter is an interesting option, since its incorporation into the catalyst enhances activity, selectivity, and stability. The promoters can be used for modifying both the active phase and the support and may therefore contribute to the following aspects: (i) improving thermal stability, as is the case of small amounts of SiO_2_ or ZrO_2_ into γ-Al_2_O_3_, which shift the phase transition from γ-Al_2_O_3_ to α-Al_2_O_3_ toward higher temperatures;^119^ (ii) hindering undesirable secondary reactions leading to catalyst deactivation by coke formation, which are attenuated by adding promoters with basic properties or capacity for gasifying the coke deposited during the reaction;^120,121^ and (iii) improving the dispersion of the active phase metal.^12,122^

#### 3.1.2 Catalyst Properties

**Figure 5:**
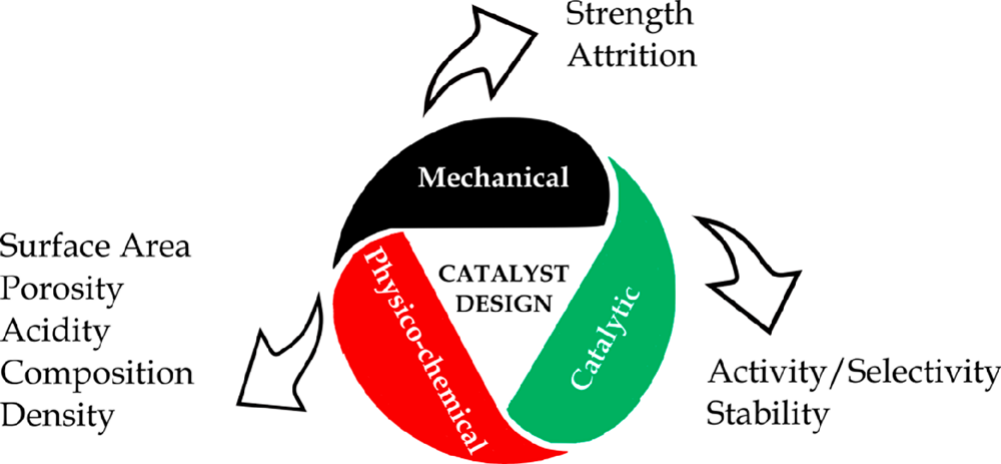
Properties involving catalyst design.

The design of reforming catalysts implies a compromise involving mechanical, physico-chemical, and catalytic properties. The balance of these interconnected properties is illustrated in Figure 5, with the relative significance of these features being greatly influenced by the process type, reactor design, process conditions, and economic factors. Thus, the selection of a suitable catalyst particle size leads to a good flow distribution and low pressure drop in the reactor for a catalyst having suitable mechanical strength and attrition resistance.

High activity and selectivity are required for the catalysts, which are mainly influenced by the chemical components making up the catalyst but also by the preparation method and synthesis conditions. Both factors (catalytic materials and synthesis method) play a key role for attaining a proper specific surface area and ensuring a good dispersion of the metal active phase onto the support. In addition, the balance between porosity and mechanical strength should be considered, since a highly porous catalyst leads to a high activity at the expense of decreasing the mechanical strength.^123^

An active catalyst fulfilling the aforementioned requirements is not enough, but stability should also be considered. Thus, catalyst lifetime depends on its resistance to deactivation mechanisms, which in reforming reactions are mainly metal sintering and coke deposition. Therefore, although thermal stability and coke resistance are sensitive to process conditions, they are usually improved by adding a promoter.^117,124^

#### 3.1.3 Synthesis Method

This section deals with the description of the usual catalyst synthesis methods in both the laboratory and industry for the preparation of heterogeneous catalysts. Thus, the preparation of an active catalyst can be carried out by different synthesis methods, and the properties obtained are strongly affected by each step in the preparation method and the quality of the raw materials.^125^

The significance of solid catalysts in large scale processes for the conversion of chemicals, fuels and pollutants is well-known. Moreover, the catalysts may be classified as follows: (i) unsupported (bulk) catalysts, (ii) supported catalysts, (iii) confined catalysts (ship-in-a-bottle catalysts), (iv) hybrid catalysts, and (v) polymerization catalysts, among others.^126^ However, with the aim of simplifying these groups, some authors have classified them according to the preparation procedure, as follows: (i) bulk catalysts or supports, in which the catalytic active phase is generated as a new solid phase; and (ii) impregnated catalysts, in which the active phase is introduced or fixed on a pre-existing solid by a process depending on the support surface.^127^ In addition, a third group was included by Hutchings and Védrine,^128^ i.e., (iii) mixed-agglomerated catalysts, in which the catalyst is an agglomerated mixture of an active substance and the support. However, this type of catalyst has been less frequently used in the literature.

**Figure 6:**
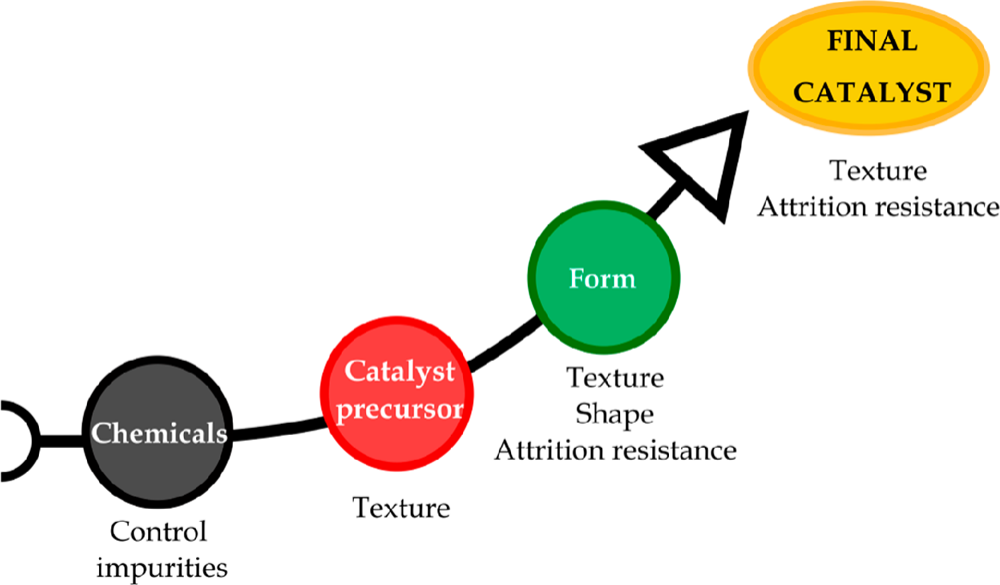
Properties acquired by catalysts through the synthesis process.

Generally, the catalyst must have certain features for each specific process, such as suitable texture (specific surface area, pore structure, and bulk density), attrition resistance, and shape, with the final properties being highly dependent on the preparation steps (Figure 6).

**Figure 7:**
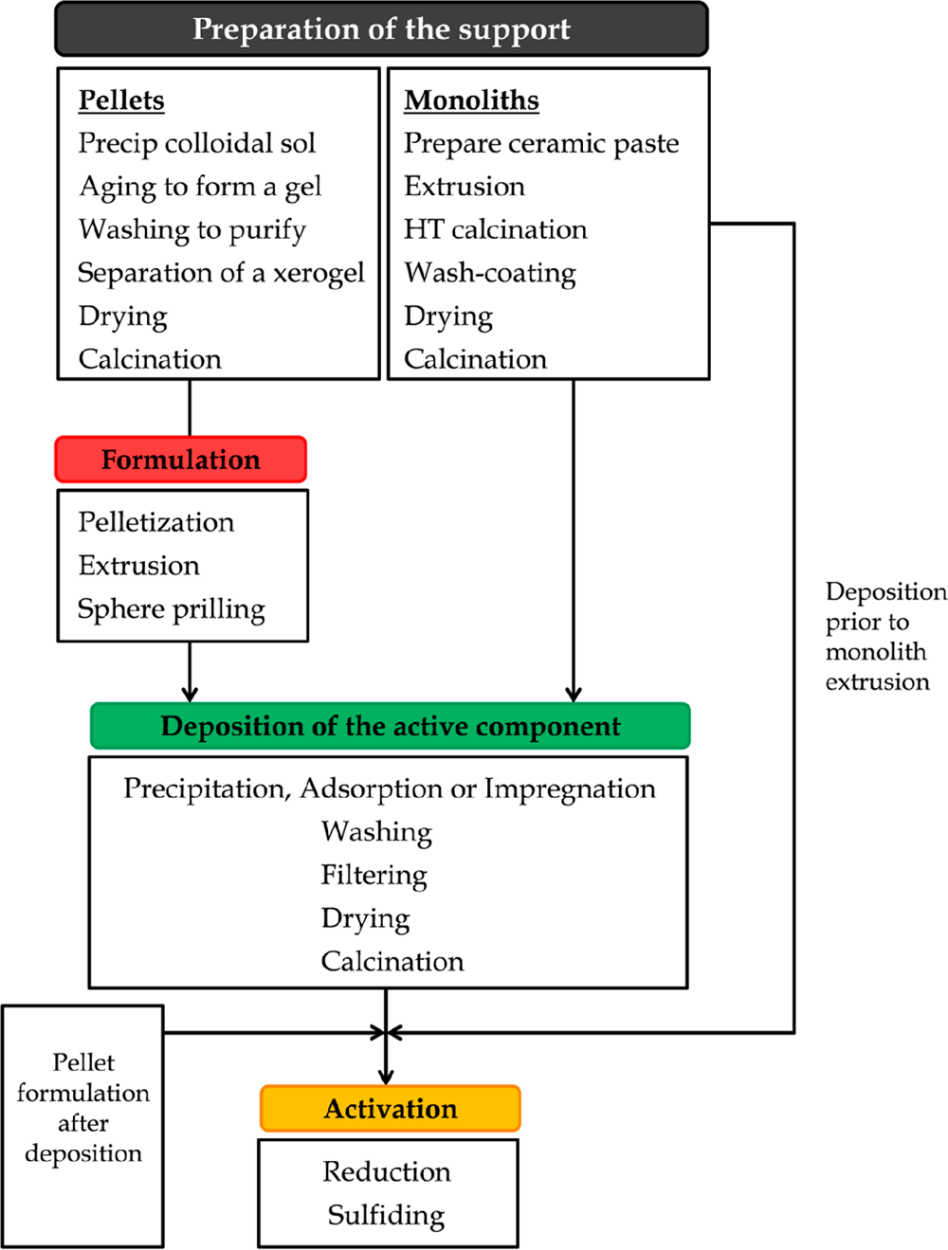
General scheme for catalyst preparation and formation.^129^

The catalysts manufactured in industry involve the following steps:^128^ (i) precipitation or other synthesis processes, e.g., sol–gel, solid–solid, flame hydrolysis, vapor deposition; (ii) hydrothermal transformation; (iii) decantation, filtration, centrifugation; (iv) washing; (v) crushing and grinding; (vi) forming and/or shaping operations; (vii) calcination; (viii) impregnation; (ix) mixing; and (x) activation, reduction. Thus, a general scheme of catalyst preparation and formation is shown in Figure 7.

As mentioned above, the catalysts may be classified into non-supported or bulk, supported, and mixed-agglomerated ones. The different types of catalysts and their preparation methods are briefly explained below.

##### 3.1.3.1 Bulk Catalysts

Bulk catalysts are mainly made up of an active substance, although some inert binder is frequently added to ease the forming and/or shaping operation. The addition of this binder only influences the mechanical strength and should therefore be considered when operating under certain conditions in specific reactors. Accordingly, bulk catalysts can be used without binder addition, with the catalysts being prepared by high-temperature fusion.

Among the methods for preparing bulk catalysts, the following ones are worth mentioning: (i) precipitation, which is the most widely used for its simplicity and cost; (ii) sol–gel, which allows the preparation of aerogels or xerogels; (iii) hydrothermal synthesis; and (iv) flame hydrolysis.

##### 3.1.3.2 Supported Catalysts

Supported catalysts have been widely used in both laboratory and large scale plants, given that they have certain advantages, such as: (i) high active phase dispersion, (ii) high mechanical strength and thermal conductivity, (iii) suitable catalytic properties by means of different metal–support interactions, and (iv) suitable for preparing bifunctional catalysts.

Moreover, some of the bulk materials previously described as unsupported catalysts can also be used as supports, with the most widely used being Al_2_O_3_, SiO_2_, TiO_2_ or ZrO_2_.^126^ Thus, the support can be synthesized following the different steps displayed in Figure 7. However, commercial preformed supports are usually considered more convenient, efficient, and economic,^116^ given that they already have the desired porous texture and mechanical strength.

Once the support with the optimum physical, chemical, and mechanical properties has been prepared or selected and stabilized with additives if required, the active phase is dispersed on the support by one of the following methods: (i) precipitation, (ii) adsorption, (iii) ion exchange, and (iv) impregnation. All these methods have certain advantages and disadvantages, and the selection of one or the other depends on the final features required for the catalyst. These methods are described below.

##### 3.1.3.2.1 Precipitation

The aim of the precipitation method is to achieve the following reaction:
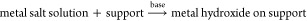
8

The choice of the salt precursor or the alkali depends on several factors, with the most influential being the possible detrimental effects on the final catalyst.^116^ Thus, the support, frequently powder, is slurried with enough amount of salt solution to reach the desired metal loading. Subsequently, enough alkali solution is added to cause precipitation, and the powder is then filtered or separated and washed to remove alkali ions, reagent anions, and an excess deposit on the outside of the particles. After washing, the excess of moisture is removed from the pores by drying, which is not a problematic step given that the active component is firmly anchored to the surface. The calcination step is then carried out to decompose the metal salt precursors into their oxidized form.

Moreover, the processes involved in the precipitation are as follows: (i) precipitation from bulk solutions or from pore fluids and (ii) interaction with the support surface. The precipitation takes place in three steps, supersaturation, nucleation, and growth, and depends on concentration, temperature, pH, and ripening.^125,127^ This method is usually preferred when loadings higher than 10–20% are required.

##### 3.1.3.2.2 Adsorption

In this process, the support is exposed to metal salt solutions in which equilibrium amounts of salt ions are adsorbed following adsorption isotherms. Depending on the properties of the support surface, the adsorption can be cationic or anionic. This process is greatly influenced by the adsorption conditions, especially by the pH of the solution.^126^

The equilibrium reactions involved in the ionic adsorption (except for zeolites) are as follows:
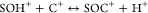
9
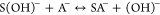
10

Moreover, the support (as powder or particle form) is dehydrated and soaked into the solution for a suitable period of time, leading to a uniform active phase distribution, with the pores being properly filled during the soaking time. Likewise, drying and calcination steps are required.

Therefore, although this is an excellent method when low metal loadings are required, the amount of metal loading allowed until the saturation point is generally small (e.g., loadings of 2–3 wt % of Ni on Al_2_O_3_ are obtained).^116^

##### 3.1.3.2.3 Ion Exchange

The catalyst synthesis by ion exchange is very similar to ionic adsorption. However, it involves the exchange of an ion by electrostatic interaction with the support surface by another ion species.^125,127^ The support containing ions A is plunged into an excess volume (compared to the pore volume) of a solution containing ions B, which gradually penetrate into the pores of the support, while ions A pass into the solution until equilibrium is reached, according to the following reaction:^130^
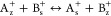
11with s and z being the solution and the support, respectively. This is an adequate method for removing harmful agents and adding promoters and is considered as a promising alternative for the modification of catalytic materials.^116^

##### 3.1.3.2.4 Impregnation

Impregnation is the simplest and most direct method to deposit the active phase onto the support. It implies the contact between a certain volume of active phase metal precursor and the solid support, with the solvent being removed in a subsequent drying step. This methodology allows improving the metal dispersion on the support, although the selection of the suitable metal salt precursor also plays a key role in the final dispersion.^131^

Depending on the volume of the solution, the impregnation may be classified as follows: (i) wet impregnation, in which an excess of solution is used, and (ii) incipient wetness impregnation, in which the volume of a solution with appropriate concentration is equal or slightly lower than the pore volume of the support.

In this method, the solubility of the precursor in the solution limits the maximum metal loading, although another impregnation step may be conducted after the drying or calcination step when higher metal loadings are required.

Furthermore, the drying process, which is necessary to crystallize the salt on the pore surface, may lead to irregular and uneven concentration, and this step should therefore be slow enough to form uniform deposits. Moreover, the calcination step, in which the salt precursor is converted into its oxide form, also plays a key role in the final metal dispersion.^116^

##### 3.1.3.3 Physical Mixing

Mixed agglomerated catalysts are prepared by this method. These catalysts are prepared by physically mixing the active substances with a powdered support or support precursors in a ball mill. The final mixture is then agglomerated and activated.

The selection of a given preparation method and its synthesis steps condition the catalyst properties and therefore its overall performance in the pyrolysis-reforming step. In order to analyze their influence in detail, these studies should be performed under similar operating conditions. Although few research studies have investigated the influence the synthesis method and conditions have on the reforming step, the main studies are summarized and discussed in section 4.

### 3.2 Mechanism of Catalyst Deactivation

Over the last decades, the mechanisms and causes of catalyst activity decay have been widely analyzed in order to step further into the knowledge of catalysis, thereby establishing the basis for modeling deactivation processes, improving catalyst design, and preventing or slowing the degradation of the catalyst.^116,123,129,132,133^

The economic feasibility of a given catalytic process largely depends on the activity and catalyst lifetime, and although the catalyst activity decay with time is unavoidable, certain strategies may be developed in order to improve its performance during the reaction step. Thus, possible causes of catalyst deactivation and their mechanisms are described below.

The causes of catalyst deactivation can be ascribed to three main factors: (i) mechanical, (ii) thermal, and (iii) chemical. However, deactivation is not the consequence of a single mechanism, but usually their combination is responsible for the catalyst degradation. Table 2 shows the main causes of catalyst activity decay. As observed, the mechanisms may be classified as follows: (i) poisoning, (ii) coking and fouling, (iii) sintering, (iv) component volatilization, (v) inactive compound formation, (v) phase transformation, and (vi) particle failure or attrition.

**Table 2:** Causes of Catalyst Deactivation

Mechanism	Type	Result
Poisoning	Chemical	Loss of active sites
Fouling/coking	Mechanical/chemical	Loss of surface, plugging
Sintering	Thermal	Loss of surface
Component volatilization	Thermal/chemical	Loss of catalytic phases
Inactive compound formation	Thermal/chemical	Loss of catalytic phases and surface
Phase change	Thermal	Loss of surface
Particle failure	Mechanical	Bed channeling, plugging

#### 3.2.1 Poisoning

Poisoning is the strong chemisorption of reactants, products, or impurities on sites otherwise available for catalysis.^134^ The fact that a compound acts as a poison depends on the nature of the process and the adsorption strength of this compound to physically block the active sites. Besides, the poison can lead to the modification of the catalyst structure or formation of a compound.

Moreover, the decrease in catalytic activity as a consequence of poisoning can involve the following aspects: (i) physical blockage of the adsorption/reaction sites; (ii) electronic modification of the nearest metal atoms, thus hindering their capability to adsorb and dissociate the reactants; (iii) dramatic changes in the catalytic properties by rearranging the catalyst surface; (iv) blockage of reactant access; and (v) hindrance of surface diffusion of adsorbed reactants.^123^

Most of the compounds considered as poisons are contained in the feed in small quantities and deactivate the catalyst following a different mechanism from the main reaction. However, poisons can also be generated either in parallel or in a series of reactions, resulting in catalyst activity decay.^116^

The irreversibility of poisoning and catalyst regenerability are greatly influenced by the type of poison, the catalyst, and the process. Thus, the main poison of reforming catalysts is sulfur, which may be in the feed as an organic sulfur compound, at concentrations of up to 1500 ppm.^135^ However, the catalyst tolerance against poisons depends on the materials that make up the catalyst, with the best performance against poisoning deactivation having been reported for noble metal based catalysts.^136,137^

#### 3.2.2 Fouling/Coking Deposition

The physical formation of species deposits from the fluid phase on the catalyst surface is the common mechanism of deactivation known as coking or fouling, although this latter term can also be associated with other types of deposition from the reactor material.^138^ Fouling leads to a loss of activity due to the blockage of active sites and/or pores and may involve the breakup of catalyst particles and reactor plugging.^123^

**Figure 8:**
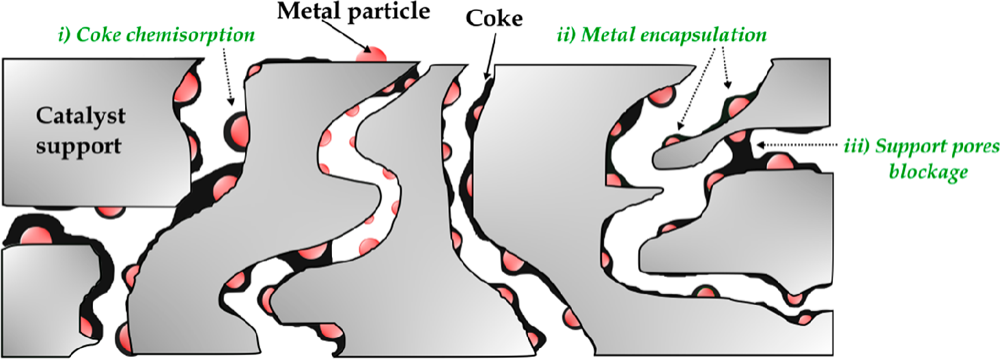
Deactivation by carbon deposition.

The consequences of carbon deposition are shown in Figure 8 and may be summarized as follows: (i) the coke deposited is strongly chemisorbed to the metal particle or physically adsorbed in a multilayer, hindering the access of reactants to the active sites; (ii) full deactivation of the metal particle due to its complete encapsulation by coke deposition; (iii) blockage of the support pores, hindering the access of the reactants to the active sites; and (iv) fracture of the support material due to the formation and growth of filaments, which may lead to reactor plugging.^134^

The mechanisms of coke formation and its intrinsic nature are greatly influenced by the feed, the type of process, and its operating conditions. Moreover, the nature of this coke may evolve with time on stream. Thus, some authors have reported that the main coke precursors are aromatics and/or olefins, which by reactions of dehydrogenation, condensation, and oligomerization, lead to coke formation.^138^

**Figure 9:**
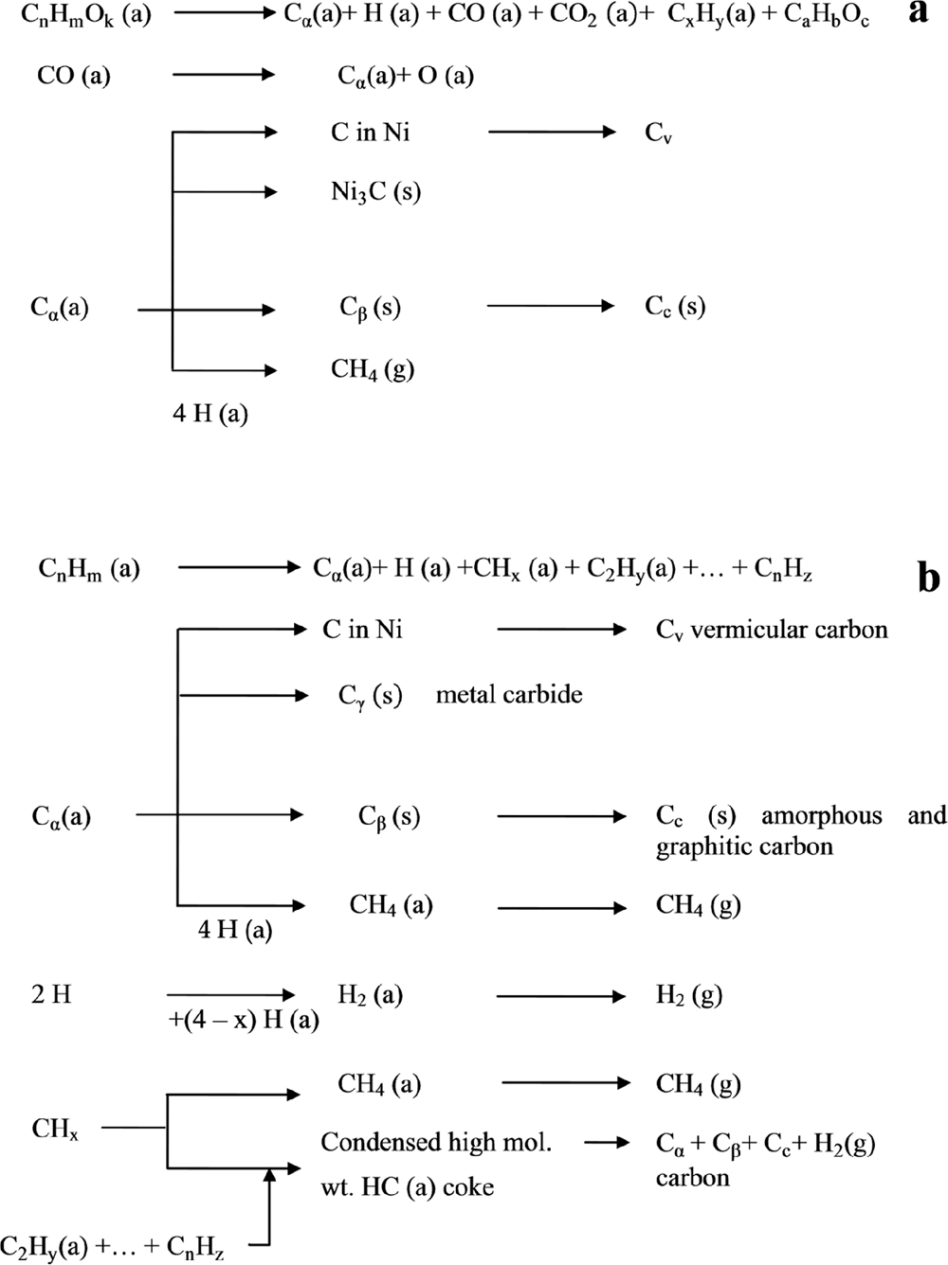
Mechanisms of coke formation and transformation during the reforming of oxygenates (C_*n*_H_*m*_O_*k*_) (a) and hydrocarbons (C_*n*_H_*m*_) (b). ((a), (g), and (s) refer to the adsorbed, gaseous, and solid states, respectively); gas phase reactions are not considered. Reproduced with permission from ref (139), Copyright 1982 Taylor & Francis Ltd. and ref (140), Copyright 2020 Elsevier.

Several authors have studied the mechanisms of carbon deposition and coke formation on metal catalysts from carbon monoxide and hydrocarbons in steam reforming reactions.^133,134,139^ Accordingly, the mechanisms proposed by Bartholomew^139^ are presented in Figure 9.

As observed, the nature and morphology of the coke formed differs depending on the different reactions described above, with its formation and nature depending on the reaction conditions (Table 3).

**Table 3:** Carbon Species Formed According to the Aforementioned Mechanisms^123^

Structural type	Designation	Temp formed (°C)
Adsorbed, atomic (surface carbide)	C_α_	200–400
Polymeric, amorphous films or filaments	C_β_	250–500
Vermicular filaments, fibers, and/or whiskers	C_v_	300–1000
Nickel carbide (bulk)	C_γ_	150–250
Graphitic (crystalline) platelets or films	C_c_	500–550

Furthermore, as mentioned above, the mechanism of catalyst deactivation and coke formation is greatly influenced by the feed. Thus, in the steam reforming of the volatiles derived from the pyrolysis of biomass and plastic wastes, the cracking of oxygenates (eq 1) and hydrocarbons, HCs (eq 2), respectively, should be taken into account.

Accordingly, Ochoa et al.^141^ analyzed the role oxygenates play in the catalyst deactivation in the steam reforming of bio-oil and distinguished between two types of coke: (i) encapsulating coke, with mainly aliphatic nature and oxygenates in its composition, since its precursors are mostly bio-oil oxygenates adsorbed on the metallic sites, and (ii) filamentous coke, with a higher ordering and carbonization in its structure, i.e., more polyaromatic and olefinic compounds, whose possible precursors are CO and CH_4_, largely contributing to a more severe catalyst deactivation. Likewise, the same authors evaluated the deactivation mechanisms of a Ni supported catalyst in the pyrolysis-steam reforming using HDPE as raw material.^142^ They reported that the mechanisms of coke formation follow two consecutive steps: (i) the formation of amorphous and encapsulating coke by condensation of promoters, and (ii) carbonization of adsorbed coke promoters leading to the formation of filamentous coke.

Thus, the formation of carbon deposits on metal oxides involves cracking reactions of the coke precursors, which are catalyzed by the acid sites, and may evolve to a more condensed coke by dehydrogenation and cyclization reactions.^123^

Several characterization techniques have been used in the literature in order to analyze catalyst and coke properties during the catalyst deactivation in the reforming step.^140^ Thus, Ochoa et al.^143^ conducted a detailed analysis of the evolution of catalyst deactivation in the biomass pyrolysis and in-line steam reforming. Thus, they determined coke content by TG-MS/TPO and analyzed its morphology by scanning and transmission electron microscopy (SEM and TEM), reporting its encapsulating nature as the main cause of catalyst decay.

SEM and TEM images have also been used in the pyrolysis-reforming of wastes plastics for analyzing the nature of the coke deposited. Thus, Barbarias et al.^46^ reported the presence of filamentous carbon in the reforming of HDPE pyrolysis volatiles, although no filamentous carbon was observed in the coke deposited when PS was used as the feedstock.

#### 3.2.3 Sintering

The agglomeration and growth of the catalyst metal crystallites is the process known as sintering, which is influenced by several factors, such as temperature and the reaction medium, among others.^135^ Thus, high temperatures, typically above 500 °C, and the presence of steam promote the sintering phenomenon, leading to irreversible or difficult to reverse deactivation of the catalyst. Besides, the metal type and its dispersion on the support, the presence of promoters or impurities on the surface, and the textural properties of the support greatly influence the sintering rates.^134^

The influence of temperature on metal sintering has been correlated as follows:
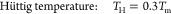
12
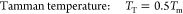
13where *T*_m_ is the melting point temperature in K. Accordingly, atomic particles on the surface, especially surface atoms weakly bound to defect sites, become mobile at temperatures above the Hüttig temperature, and bulk metal atoms acquire enough thermal energy to migrate within the crystallite at temperatures above the Tamman one.^116,138^

**Figure 10:**
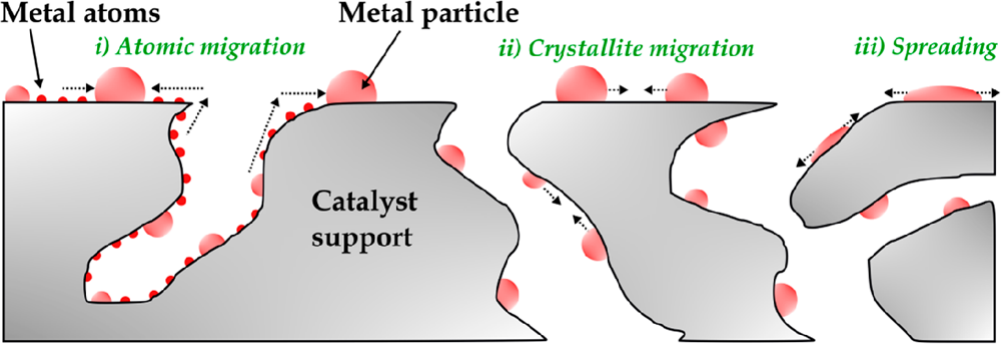
Deactivation by sintering.

The mechanisms whereby metal particles increase in size may occur as illustrated in Figure 10, and they are summarized as follows: (i) atomic migration, which implies metal atom detachment from crystallites and their migration to the support surface, where they are finally captured by larger metal particles; (ii) migration of entire crystallites, forming larger particles by collision and coalescence; and (iii) spreading and splitting.^123,134^

The catalyst activity decay by sintering may lead to: (i) crystallite growth on the catalytic phase, decreasing the catalytic surface area, and (ii) collapse of the support and/or support pores, involving a loss of catalytic surface area and loss of support area.

Moreover, the support may also be subject to sintering involving the following processes: (i) surface diffusion, (ii) solid-state diffusion, (iii) grain boundary diffusion, (iv) evaporation/condensation of volatile atoms or molecules, and (v) phase transformations.^123^ These two latter processes are explained in the following sections.

#### 3.2.4 Component Volatilization

The catalyst deactivation by direct volatilization of catalytic metals hardly plays a significant role in the catalyst activity decay, given that extremely high temperatures are required (normally, up to 1000 °C) for this process. On the contrary, the formation of volatile compounds, such as metal carbonyls, oxides, sulfides, and halides, which lead to a loss of catalytic material, can take place at moderate temperatures.^123^

Some examples of catalyst deactivation by component volatilization are found in the regeneration of molybdenum-containing catalysts in the hydrotreating process when temperatures of up to 800 °C are attained (Mo vaporizes at this temperature). It also applies in the naphtha steam reforming, although coke formation from heavier hydrocarbons is controlled in this process with potassium. Nevertheless, volatile KOH compound may be formed in the presence of steam, which accelerates coke formation.^138^

#### 3.2.5 Inactive Compound Formation

A further chemical route leading to catalyst deactivation is the reaction between the vapor phase and the catalyst surface to produce inactive phases instead of strongly adsorbed species, which lead to a decrease in catalyst activity. Moreover, these reactions are usually promoted at high temperatures and can be easily detected by the X-ray diffraction (XRD) technique.

Accordingly, the reaction between the active phase of nickel with common supports, such as Al_2_O_3_ or SiO_2_ to form nickel aluminates or silicates, the formation of oxides with steam when cobalt or iron are used,^116^ or the oxidation, sulfidation, or carbidation of the metal active phase involve the loss of catalytic activity.^134^

#### 3.2.6 Phase Transformation

Phase transformation is the consequence of the thermal degradation of the support and adversely affects physical or chemical catalyst properties. Thus, the support can be induced by thermal treatment to attain a phase modification, as in the case of the Al_2_O_3_ support, which can change from γ-Al_2_O_3_ to a transitional phase with lower specific surface area (δ-Al_2_O_3_, θ-Al_2_O_3_...) until reaching the most stable phase (α-Al_2_O_3_). Consequently, the supported catalyst surface area decreases, and therefore activity drops. Several authors have called this process support sintering.^123^ Moreover, the addition of small amounts of silica may contribute to control this phase change.^131^

#### 3.2.7 Particle Attrition

The catalyst mechanical failure can arise from (i) granule, pellet, or monolithic crush when loading; (ii) attrition or reduction/breakup of the catalyst particle size forming fine particles, especially in fluidized bed reactors; and (iii) erosion of the catalyst particles when high fluid velocities are used.^134^ As a result, reactor plugging or channeling may occur, leading to an increase in pressure drop and non-uniform bed performance. Thus, thermal and coking effects promote other catalyst deactivation mechanisms.

In order to prevent catalyst attrition, the following alternatives have been proposed in the literature: (i) increasing strength by advanced catalyst synthesis methods, (ii) adding binders to improve strength and toughness, (iii) coating aggregates with porous but very strong materials, and (iv) inducing compressive catalyst stress by chemical or thermal tempering.^123,134^

## 4 Application of Metallic Catalysts in the Pyrolysis-Reforming Process

In recent decades, the development and study of a wide range of different metal catalysts has been approached in the literature for the reforming of oxygenates derived from biomass pyrolysis. However, most of these studies have been conducted by using model compounds^144−146^ or the aqueous fraction of the bio-oil.^34,36,107^ Similarly, the valorization of different waste plastics, namely, polyolefins (HDPE, LDPE, and PP), polystyrene (PS), polyethylene terephtalate (PET), and real world plastics recovered from municipal solid waste (MSW), or even the joint co-feeding of biomass and plastics mixtures, has attracted increasing attention in the two-step pyrolysis-reforming process in recent years due to the great versatility of this strategy.^54,56,147,148^ Furthermore, special attention has been paid to the design of a suitable reforming catalyst for the production of H_2_. This section reviews the catalysts used in the literature for the two-step processing of biomass, plastic wastes, and biomass/plastics mixtures, in which pyrolysis is carried out in the first step and in-line steam reforming of the volatiles in the second one.

Accordingly, and with the aim of easing the comparison of the different catalysts, the following sections have been considered: (i) Ni/Al_2_O_3_ catalysts, which are the most widely used because Al_2_O_3_ support provides a high specific surface area, eases Ni dispersion, and confers mechanical strength and stability upon the catalyst^149^ (section 4.1); (ii) Ni supported on other materials, either on several metal oxides, such as SiO_2_, MgO, ZrO_2_, or TiO_2_ or on other types of supports, such as chars obtained from the fast pyrolysis of different biomasses, dolomite, chicken dropping (CD), or pig manure compost (PC), among others (section 4.2); (iii) Ni/Al_2_O_3_ catalysts modified with different promoters, such as CeO_2_, MgO or alkali compounds, which improve the properties of the bare catalyst and therefore enhance its activity or stability (section 4.3); and (iv) bimetallic and non-Ni based catalysts containing alternative active phases, such as Fe, Co, or Cu, or even noble metals, such as Rh, Pd, Ru, or Pt, which are generally more active than Ni (section 4.4).

**Figure 11:**
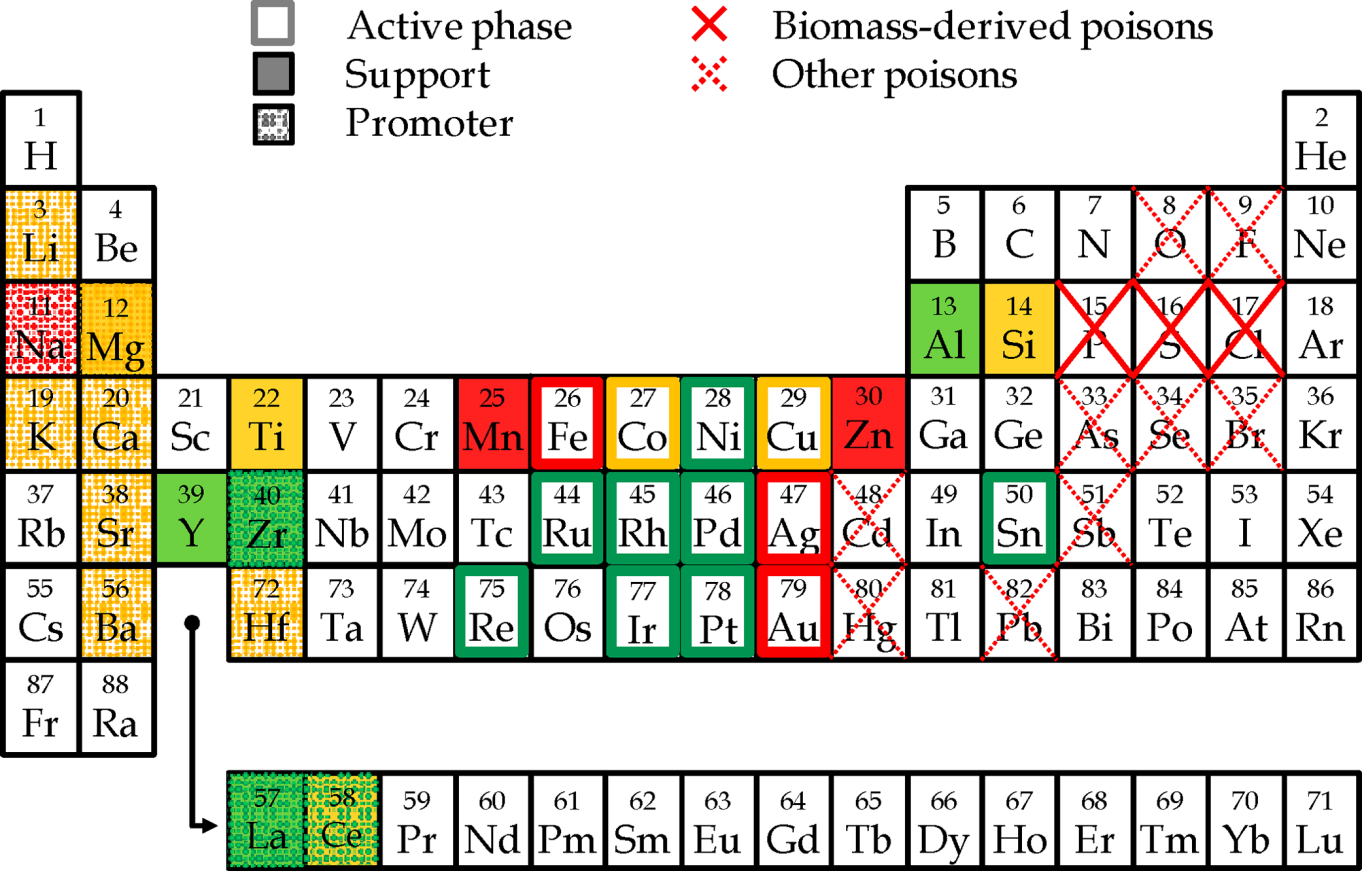
Main elements studied in the catalyst design for H_2_ production from the pyrolysis and in-line steam reforming process using biomass, plastic wastes, and biomass/plastic mixtures as feedstock. Adapted from ref (12). Copyright 2009 American Chemical Society.

Figure 11 shows the main components used as catalytic materials, namely, the active phase, promoter, or support, in the catalyst design for H_2_ production from the steam reforming of the volatiles derived from the pyrolysis of biomass, plastic wastes, and their mixtures. Thus, the suitability of these elements has been classified according to a color code, with green, yellow, and red being good, moderate, and poor performances, respectively.

### 4.1 Ni/Al_2_O_3_ Catalysts

Ni/Al_2_O_3_ catalysts (both commercial and prepared) have been widely studied in the literature on the reforming reactions.^50,110,150,151^ Thus, Ni has proven to be highly active on the reforming of the volatiles derived from biomass and plastic wastes pyrolysis as well as on methane steam reforming, WGS reaction, and ammonia decomposition.^11,152,153^ Moreover, its moderate cost made this metal an interesting option compared to other active phases, such as noble metals (Rh, Ru, Pd, Pt...). Similarly, Al_2_O_3_ is the most widely used support in reforming reactions, and its suitable properties (e.g., high specific surface area) allow an adequate Ni dispersion and provide stability and mechanical strength upon the catalyst, making this support suitable for fluidized bed reactor configurations.^149^ Nonetheless, catalysts supported on Al_2_O_3_ undergone a severe deactivation by carbon deposition, which is promoted by the acid properties of this support.^100,117^

Regarding the crystalline phases of the Al_2_O_3_ support, significant differences in the catalyst properties can be observed. Thus, the transition from γ-Al_2_O_3_ to α-Al_2_O_3_, which is usually formed at extremely high temperatures (>1200 °C) through intermediate crystal phases such as θ- and δ-Al_2_O_3_, leads to a remarkable decrease in the specific surface area of the support and therefore to a poorer active phase dispersion on the catalyst. Furthermore, the metal–support interaction is also influenced by the type of Al_2_O_3_ selected, and the reducibility of the metal catalyst is therefore modified, i.e., weaker interaction between metal and α-Al_2_O_3_ is obtained in comparison with γ-Al_2_O_3_, which is more likely to form a spinel phase.^154^ Besides, α-Al_2_O_3_ has proven to be attrition resistant when operating in fluidized bed reactors.^155^

Moreover, the acidity of the support is related to the crystalline phase of the Al_2_O_3_ support. Thus, the acid sites of γ-Al_2_O_3_ promote carbon deposition on the catalyst surface, which is the main cause of catalyst deactivation in reforming reactions.

As mentioned before, the operating conditions, type of reactor configuration, and the catalyst selected in the pyrolysis-reforming strategy greatly influence the overall H_2_ production. Accordingly, Tables 4–6 summarize the main results reported in the literature for Ni/Al_2_O_3_ catalyst for the pyrolysis-steam reforming process of biomass, plastic wastes, and biomass/plastics mixtures. In regards to the use of biomass as raw material (Table 4), an extensive use of commercial catalysts has been reported in the literature. Xiao et al.^61^ carried out a parametric study in a two-step fluidized bed/fixed bed configuration, using wood chips and pig manure compost as feedstock and Ni/Al_2_O_3_ and Ni/BCC (brown coal char) as catalysts. Although Ni/Al_2_O_3_ undergoes gradual catalyst deactivation, mainly by carbon deposition, wood chip biomass leads to higher H_2_ productions compared to pig manure compost (7.2 and 5.0 wt %, respectively, on dry and ash free, daf).

**Table 4:** Ni/Al_2_O_3_ Catalysts Reported in the Literature for the Pyrolysis and In-line Steam Reforming of Biomass

Catalyst	Feed	Preparation method^a^	Reactor configuration	Operating conditions^c^	H_2_ conc. (vol %)	H_2_ prod. (wt %)	Ref.
20Ni/Al_2_O_3_	pine wood	commercial	fluidized/fixed (1–2 g min^–1^)	*T*_P_ = 530–700 °C	60.0	7.2^b^	Xiao et al.^61^
				*T*_R_ = 550–710 °C			
20Ni/Al_2_O_3_	pig manure compost	commercial	fluidized/fixed (1–2 g min^–1^)	*T*_P_ = 530–700 °C	56.2	5.0^b^	Xiao et al.^61^
				*T*_R_ = 550–710 °C			
11Ni/Al_2_O_3_	pine wood sawdust	commercial (G90 LDP)	spouted/fluidized (0.75 g min^–1^)	*T*_P_ = 500 °C	66.0	11.2	Arregi et al.^50^
				*T*_R_ = 550–700 °C			
				S/C = 7.7			
11Ni/Al_2_O_3_	pine wood sawdust	commercial (G90 LDP)	spouted/fixed (0.75 g min^–1^)	*T*_P_ = 500 °C	64.5	10.5	Fernandez et al.^98^
				*T*_R_ = 600 °C			
				S/C = 7.7			
10Ni/Al_2_O_3_	wood sawdust	commercial	fixed/fixed (1 g)	*T*_P_ = 300–600 °C	38.1	2.2	Olaleye et al.^158^
				*T*_R_ = 800 °C			
20Ni/Al_2_O_3_	sewage sludge	commercial	fixed/fixed	*T*_P_ = 900 °C	70.0	11.6^b^	Cao et al.^156^
				*T*_R_ = 400–750 °C			
20Ni/Al_2_O_3_	Japanese cypress	commercial	fixed/fixed (1 g)	*T* = 450 °C	35.2	5.0^b^	Kannari et al.^159^
12Ni/Al_2_O_3_	cedar wood	iwi	dual fixed bed (0.06 g min^–1^)	*T* = 550–650 °C	60.1	8.3	Miyazawa et al.^89^
				S/C = 0.5			
20Ni/Al_2_O_3_	wood pellets	iwi	screw-kiln/fixed (4 g min^–1^)	*T*_P_ = 500 °C	44.4	2.4	Efika et al.^102^
				*T*_R_ = 760 °C			
10Ni/Al_2_O_3_	pine wood sawdust	wi	spouted bed/fluidized (0.75 g min^–1^)	*T*_P_ = 500 °C	64.2	10.2	Santamaria et al.^51,100^
				*T*_R_ = 600 °C			
				S/C = 7.7			
3Ni/Al_2_O_3_	pine sawdust	wi	entrained-flow/fixed (4 g min^–1^)	*T*_P_ = 900 °C	49.3	6.1	Liu et al.^160^
				*T*_R_ = 800 °C			
15Ni/Al_2_O_3_	cellulose	wi	fixed/fixed (1.5 g)	*T*_P_ = –		5.9	Zou et al.^161^
				*T*_R_ = 800 °C			
10Ni/Al_2_O_3_	rice husk	iwi	fixed/fixed (2 g)	*T*_P_ = 550 °C	57.6	3.7	Akubo et al.^157^
				*T*_R_ = 750 °C			
10Ni/Al_2_O_3_	coconut shell	iwi	fixed/fixed (2 g)	*T*_P_ = 550 °C	58.2	4.4	Akubo et al.^157^
				*T*_R_ = 750 °C			
10Ni/Al_2_O_3_	sugar cane	iwi	fixed/fixed (2 g)	*T*_P_ = 550 °C	59.2	4.6	Akubo et al.^157^
				*T*_R_ = 750 °C			
10Ni/Al_2_O_3_	palm kernel shell	iwi	fixed/fixed (2 g)	*T*_P_ = 550 °C	57.4	5.1	Akubo et al.^157^
				*T*_R_ = 750 °C			
10Ni/Al_2_O_3_	cotton stalk	iwi	fixed/fixed (2 g)	*T*_P_ = 550 °C	58.0	4.1	Akubo et al.^157^
				*T*_R_ = 750 °C			
10Ni/Al_2_O_3_	wheat straw	iwi	fixed/fixed (2 g)	*T*_P_ = 550 °C	54.1	3.3	Akubo et al.^157^
				*T*_R_ = 750 °C			
9Ni/Al_2_O_3_	rice husk	wi	drop-tube fixed bed (120 mg)	*T* = 800 °C	32.8	1.2	Kuchonthara et al.^103^
10Ni/Al_2_O_3_	pine wood	wi	fixed/nonthermal plasma (1 g)	*T*_P_ = 600 °C		0.8	Blanquet et al.^162^
				*T*_R_ = 250 °C			
				steam = 2 g h^–1^			

a iwi, Incipient wetness impregnation; wi, wetness impregnation.

b H_2_ production defined as g_H2_/100 g_biomass, daf_ (dry and ash free).

c *T*_P_ = pyrolysis temperature; *T*_R_ = reforming temperature.

Moreover, Arregi et al.^50^ studied H_2_ production by continuous fast pyrolysis (500 °C) of pine wood sawdust in a conical spouted bed reactor (CSBR) followed by in-line steam reforming of the pyrolysis vapors in a fluidized bed reactor. A commercial Ni/Al_2_O_3_ catalyst was highly active, with a H_2_ production of 11.2 g_H2_ 100 g_biomass_^–1^ at a reforming temperature of 600 °C, a space time of 20 g_cat_ min g_volatiles_^–1^, and a S/B ratio of 4. A similar H_2_ production was obtained by Fernandez et al.^98^ under the same operating conditions but using a CSBR and a fixed bed reactor for the pyrolysis and reforming steps, respectively. Despite this latter configuration led to lower catalyst deactivation, especially when low space times were used, the advantages of fluidized beds should be taken into account for further scalability of this strategy.

Furthermore, Cao et al.^156^ evaluated the performance of a commercial Ni/Al_2_O_3_ catalyst on the low-temperature catalytic reforming of volatiles and nitrogen compounds from sewage sludge (SS) pyrolysis in a two-step fixed bed reactor. They observed that although the results significantly depend on the operating conditions, a H_2_-rich gas stream with a content of 68.0 vol % and a H_2_ production of 11.6 wt % (daf) were obtained at 650 °C.

Besides, the synthesis of the Ni/Al_2_O_3_ catalysts has been analyzed in detail in the literature. Thus, Akubo et al.^157^ prepared a 10 wt % Ni based Al_2_O_3_ catalyst by incipient wetness impregnation and investigated the pyrolysis-catalytic steam reforming of six agricultural biomass waste samples (rice husk, coconut shell, sugar cane bagasse, palm kernel shell, cotton stalk, and wheat straw) in a two-step fixed bed reactor. The results for the different types of biomasses revealed that H_2_ production ranged from 3.3 wt % for wheat straw to 5.1 wt % for palm shell kernel. Efika et al.^102^ analyzed the performance of different prepared catalysts (Ni/Al_2_O_3_, Ni/CeO_2_-Al_2_O_3_ and Ni/SiO_2_) in a two-step continuous screw-kiln reactor, in which biomass pyrolysis (500 °C) and the subsequent catalytic steam reforming of the pyrolysis oils and gases was conducted (760 °C). They found that Ni/Al_2_O_3_ catalyst was the most effective one for H_2_ production, i.e., it allowed obtaining the highest H_2_ concentration of 44.4 vol %. Moreover, they characterized the coke deposited on this deactivated catalyst by SEM images and reported the presence of filamentous carbon. Santamaria et al.^51,100^ obtained a H_2_ production of 10.2 wt % in a CSBR-fluidized bed reactor configuration with pinewood sawdust being continuously fed at a rate of 0.75 g min^–1^ and using a 10Ni/Al_2_O_3_ catalyst prepared by the wet impregnation method.

Likewise, Ni/Al_2_O_3_ has been widely selected for waste plastic valorization by pyrolysis-reforming runs due to the aforementioned advantages, i.e., suitable activity, moderate cost, high specific surface area, good metal dispersion, and appropriate mechanical strength. Thus, Table 5 shows the main studies dealing with the pyrolysis-reforming of different waste plastics using Ni/Al_2_O_3_ as a reforming catalyst. The initial studies reported in the literature were performed by Czernik and French,^54^ who developed a continuous process consisting of two fluidized bed reactors. A commercial Ni reforming catalyst (C11-NK) used in industry for naphtha reforming was selected and PP was continuously fed at a rate of 1 g min^–1^. Operating at pyrolysis and reforming temperatures of 650 and 800 °C, respectively, these authors reported a H_2_ production of 34 wt %. A similar H_2_ production was reported by Erkiaga et al.,^97^ who conducted the continuous HDPE pyrolysis and in-line steam reforming in a bench scale plant consisting of a conical spouted bed and a fixed bed reactor for the pyrolysis and reforming steps, respectively, and using a commercial Ni based catalyst (G90-LDP). Under the optimum operating conditions, i.e., pyrolysis and reforming temperatures of 500 and 700 °C, respectively, and a S/C ratio of 3.1, they reported a H_2_ production of 34.5 wt %. However, due to the operational problems related to coke formation, Barbarias et al.^44^ conducted a parametric study in the same experimental unit but using a fluidized bed reactor instead of a fixed one. Thus, they reported a H_2_ production of 38.1 wt % at a reforming temperature of 700 °C and with a S/C ratio of 3.89. The good performance of this technology was further demonstrated in a later study on the valorization of different plastic wastes (PP, PET, and PS) and their mixtures.^56^ In this latter study, the highest H_2_ production was obtained with polyolefin plastics (37.3 and 34.8 wt % for HDPE and PP, respectively). However, a considerably lower H_2_ production was reported with PET (18.2 wt %), which is evidence of the high influence of the volatile composition and therefore of the plastic type selected.

**Table 5:** Ni/Al_2_O_3_ Catalysts Reported in the Literature for the Pyrolysis and In-line Steam Reforming of Plastic Wastes

Catalys	Feed	Preparation method^a^	Reactor configuration	Operating conditions^b^	H_2_ conc. (vol %)	H_2_ prod. (wt %)	Ref.
Ni/Al_2_O_3_	PP	commercial C11-NK	fluidized/fluidized (1 g min^–1^)	*T*_P_ = 600 °C	70.0	34.0	Czernik and French^54^
				*T*_R_ = 850 °C			
				S/C = 4.6			
11Ni/Al_2_O_3_	HDPE	commercial (G90 LDP)	spouted/fixed (0.75 g min^–1^)	*T*_P_ = 500 °C	71.0	34.5	Erkiaga et al.^97^
				*T*_R_ = 700 °C			
				S/C = 3.1			
11Ni/Al_2_O_3_	HDPE	commercial (G90 LDP)	spouted/fluidized (0.6 g min^–1^)	*T*_P_ = 500 °C	72.7	38.1	Barbarias et al.^44^
				*T*_R_ = 700 °C			
				S/C = 3.9			
11Ni/Al_2_O_3_	HDPE	commercial (G90 LDP)	spouted/fluidized (0.75 g min^–1^)	*T*_P_ = 500 °C	69.8	37.3	Barbarias et al.^56,99^
				*T*_R_ = 700 °C			
				S/C = 3.1			
11Ni/Al_2_O_3_	PP	commercial (G90 LDP)	spouted/fluidized (0.75 g min^–1^)	*T*_P_ = 500 °C	70.1	34.8	Barbarias et al.^56^
				*T*_R_ = 700 °C			
				S/C = 3.1			
11Ni/Al_2_O_3_	PS	commercial (G90 LDP)	spouted/fluidized (0.75 g min^–1^)	*T*_P_ = 500 °C	65.4	29.1	Barbarias et al.^46,56^
				*T*_R_ = 700 °C			
				S/C = 2.9			
11Ni/Al_2_O_3_	PET	commercial (G90 LDP)	spouted/fluidized (0.75 g min^–1^)	*T*_P_ = 500 °C	63.6	18.2	Barbarias et al.^56^
				*T*_R_ = 700 °C			
				S/C = 4.3			
11Ni/Al_2_O_3_	HDPE 48 wt %; PP, 35 wt %; PS, 9 wt %; PET: 8 wt %	commercial (G90 LDP)	spouted/fluidized (0.75 g min^–1^)	*T*_P_ = 500 °C	63.9	32.5	Barbarias et al.^56^
				*T*_R_ = 700 °C			
				S/C = 3.2			
10Ni/Al_2_O_3_	PP	wi	spouted/fluidized (0.75 g min^–1^)	*T*_P_ = 500 °C	70.8	34.1	Arregi et al.^55^
				*T*_R_ = 700 °C			
				S/C = 3.1			
Ni/Al_2_O_3_ (1:1 molar ratio)	PP	cp	fixed/fixed (1 g)	*T*_P_ = 500 °C	66.6	24.8	Wu and Williams^150^
				*T*_R_ = 800 °C			
				steam = 4.74 g h^–1^			
10Ni/Al_2_O_3_	PP	wi	fixed/fixed (1 g)	*T*_P_ = 500 °C	56.3	11.5	Wu and Williams^164^
				*T*_R_ = 800 °C			
				steam = 4.74 g h^–1^			
10Ni/Al_2_O_3_	PE	wi	fixed/nonthermal plasma (1 g)	*T*_P_ = 500 °C		0.9	Aminu et al.^165^
				*T*_R_ = 250 °C			
				steam = 4 g h^–1^			
10Ni/Al_2_O_3_	HDPE	wi, cp, sg	fixed/fixed (1 g)	*T*_P_ = 500 °C	62.0	12.1	Yao et al.^63^
				*T*_R_ = 800 °C			
				steam = 6 g h^–1^			
10Ni/Al_2_O_3_	PP	wi, cp, sg	fixed/fixed (1 g)	*T*_P_ = 500 °C	59.4	13.4	Yao et al.^63^
				*T*_R_ = 800 °C			
				steam = 6 g h^–1^			
10Ni/Al_2_O_3_	PS	wi, cp, sg	fixed/fixed (1 g)	*T*_P_ = 500 °C	57.9	12.5	Yao et al.^63^
				*T*_R_ = 800 °C			
				steam = 6 g h^–1^			
5 Ni/Al_2_O_3_	PP	wi	fixed/fixed (1 g)	*T*_P_ = 500 °C	49.5	6.9	Acomb et al.^163^
				*T*_R_ = 800 °C			
				steam = 4.74 g h^–1^			
5 Ni/Al_2_O_3_	LDPE	wi	fixed/fixed (1 g)	*T*_P_ = 500 °C	53.1	9.2	Acomb et al.^163^
				*T*_R_ = 800 °C			
				steam = 4.74 g h^–1^			
5 Ni/Al_2_O_3_	PS	wi	fixed/fixed (1 g)	*T*_P_ = 500 °C	60.0	7.4	Acomb et al.^163^
				*T*_R_ = 800 °C			
				steam = 4.74 g h^–1^			

a wi, Wetness impregnation; cp, co-precipitation; sg, sol–gel; ie, ion-exchange.

b *T*_P_ = Pyrolysis temperature; *T*_R_ = reforming temperature.

A two-step fixed bed configuration operating in batch mode has been widely used in the literature. Thus, the group headed by Prof. Williams investigated the effect of operating conditions, type of plastic, and catalyst synthesis method using a wide range of Ni-based catalysts.^63,150,163^ Thus, Ni/Al_2_O_3_ catalysts with different metal molar ratios (Ni/Al of 1:4, 1:2, and 1:1) synthesized by co-precipitation method were analyzed by Wu and Williams^150^ in the pyrolysis–reforming of PP at a pyrolysis temperature of 500 °C and a reforming one of 800 °C. They reported an increase in the potential H_2_ production from 48.8 to 57.7 wt % when the Ni/Al molar ratio was increased from 1:4 to 1:1, which corresponds to a rise in the H_2_ production from 20.9 to 24.8 g H_2_ per 100 g_PP_. Yao et al.^63^ investigated the influence the synthesis method has on the physicochemical properties and therefore on the catalyst activity for the production of H_2_ from pyrolysis-steam reforming of waste plastics (HDPE, PP, and PS). Accordingly, a Ni/Al_2_O_3_ catalyst was prepared by three different methods: co-precipitation, impregnation, and sol–gel. These authors observed that the catalyst prepared by the sol–gel method had higher specific surface area and fine nickel particle size with uniform dispersion, which led to higher H_2_ productions (12.1, 13.4, and 12.5 wt % for HDPE, PP, and PS samples, respectively).

**Figure 12:**
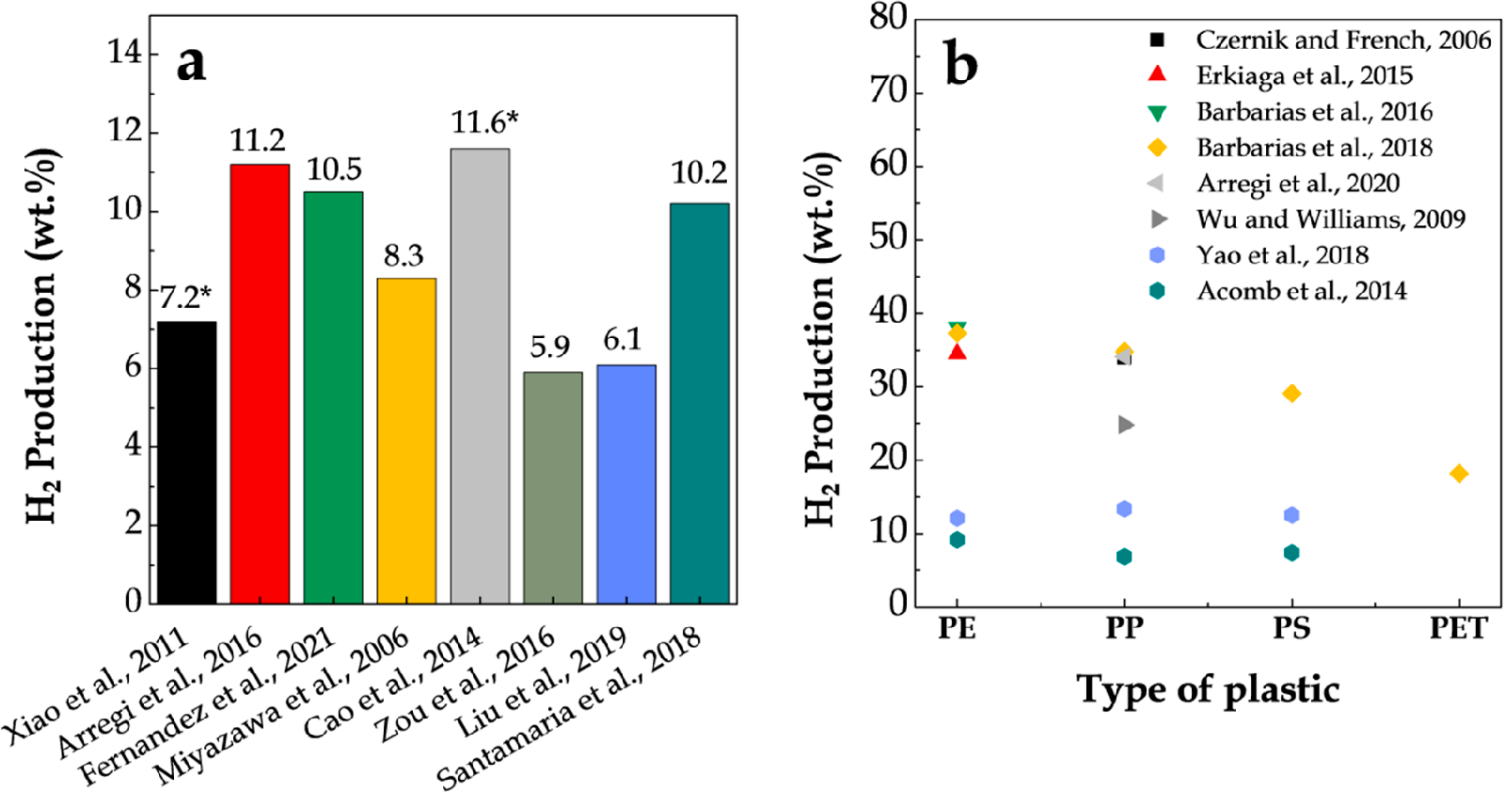
Performance of Ni/Al_2_O_3_ catalyst on H_2_ production in the pyrolysis-reforming of biomass (a) and different types of plastics (b). Xiao et al., 2011;^61^ Arregi et al., 2016;^50^ Fernandez et al., 2021;^98^ Miyazawa et al., 2006;^89^ Cao et al., 2014;^156^ Liu et al., 2019;^160^ Santamaria et al., 2018;^51^ Czernik and French, 2006;^54^ Erkiaga et al., 2015;^97^ Barbarias et al., 2016;^44^ Barbarias et al., 2018;^56^ Arregi et al., 2020;^55^ Wu and Williams, 2009;^150^ Yao et al., 2018;^63^ and Acomb et al., 2014.^163^ * corresponds to H_2_ production on a daf (dry and ash free basis).

Figure 12 compares the main results obtained in the literature for the steam reforming of the volatiles derived from the pyrolysis of biomass (Figure 12a) and different plastic wastes (Figure 12b) using Ni/Al_2_O_3_ as the reforming catalyst.

As observed in Figure 12, this catalyst shows a great potential for the production of H_2_ in the two-step pyrolysis reforming process. Thus, a H_2_ production in the range of 5.9–11.6 wt % is obtained when biomass is used as the raw material.^50,51,98,156^ Similarly, this catalyst shows an excellent performance when different types of plastic waste materials are used, being able to reach a H_2_ production of around 34 wt % when PP and PE are selected.^54,56,97^ However, the acid nature of the Al_2_O_3_ support promotes the coke formation on the catalyst, leading to a fast catalyst deactivation in the reforming step. In this regard, studies dealing with the main mechanisms of catalyst deactivation and coke formation are attracting increasing attention in the literature^140,142,143^ in order to take a step further in the scalability of the process.

A comparison of the different results reported in the literature studies is not straightforward, since they have been conducted under different reaction conditions. Thus, the reactor configuration, operating conditions (pyrolysis and reforming temperatures, steam/feedstock ratio, space time) and the catalysts synthesis conditions (preparation method, Ni loading, calcination temperature) should be taken into account for the final optimization of this process.

Moreover, the main differences observed in Figure 12 among the research studies are ascribed to the operation in continuous/discontinuous mode. Thus, the preliminary character of some of these studies conducted in laboratory-scale batch reactors led to lower H_2_ productions. In this regard, a great effort has been made for the implementation of a continuous feeding system in the two-step pyrolysis-reforming process in order to advance in the scaling up of this strategy.

The joint valorization of biomass and waste plastics on a Ni/Al_2_O_3_ catalyst in the two-step pyrolysis-reforming process has also been assessed in the literature. Thus, the co-feeding of plastics along with biomass can provide the following advantages to the process:^57,147^ (i) improvement of the overall H_2_ production by incorporating higher amount of H_2_ in the feed; (ii) limitations derived of seasonal availability of biomass are avoided; (iii) reduction of environmental problems related to the management of plastic wastes; and (iv) attenuation of catalyst deactivation in the reforming step. Accordingly, Table 6 summarizes the main studies concerning the pyrolysis and in-line catalytic reforming of biomass and plastic waste mixtures.

**Table 6:** Ni/Al_2_O_3_ Catalysts Reported in the Literature for the Pyrolysis and In-line Steam Reforming of Biomass and Plastic Mixtures

Catalyst	Feed	Preparation method^a^	Reactor configuration	Operating conditions^b^	H_2_ conc. (vol %)	H_2_ prod. (wt %)	Ref.
11Ni/Al_2_O_3_	pine wood sawdust HDPE 0, 25, 50, 75, 100	commercial (G90 LDP)	spouted bed/fluidized (0.75 g min^–1^)	*T*_P_ = 500 °C	71.1	24.6	Arregi et al.^57^
				*T*_R_ = 700 °C			
				S/(B + P) = 4			
10Ni/Al_2_O_3_	rice husk (RH) PE 0, 25, 50, 75, 100	wi	fixed/fixed (1 g)	*T*_P_ = 600 °C	46.0	4.4	Xu et al.^148^
				*T*_R_ = 800 °C			
				steam = 2 mL h^–1^			
10Ni/Al_2_O_3_	pine wood sawdust PP B/P = 80/20	wi	fixed/fixed (2 g)	*T*_P_ = 600 °C	52.1	5.5	Alvarez et al.^147^
				*T*_R_ = 800 °C			
				Steam = 4.74 mL h^–1^			
10Ni/Al_2_O_3_	pine wood sawdust HDPE B/P = 80/20	wi	fixed/fixed (2 g)	*T*_P_ = 600 °C	52.2	5.1	Alvarez et al.^147^
				*T*_R_ = 800 °C			
				steam = 4.74 mL h^–1^			
10Ni/Al_2_O_3_	pine wood sawdust PS B/P = 80/20	wi	fixed/fixed (2 g)	*T*_P_ = 600 °C	49.2	4.0	Alvarez et al.^147^
				*T*_R_ = 800 °C			
				steam = 4.74 mL h^–1^			
10Ni/Al_2_O_3_	pine wood sawdust real plastics (RP) B/P = 80/20	wi	fixed/fixed (2 g)	*T*_P_ = 600 °C	51.3	4.4	Alvarez et al.^147^
				*T*_R_ = 800 °C			
				steam = 4.74 mL h^–1^			
10Ni/Al_2_O_3_	HDPE-pine sawdust (5:5)	cp	fixed/fixed (0.5 g)	*T*_P_ = 800 °C	59.8	6.4	Chai et al.^166^
				*T*_R_ = 700 °C			
				steam = 5 mL h^–1^			
10Ni/Al_2_O_3_	PP-pine sawdust (5:5)	cp	fixed/fixed (0.5 g)	*T*_P_ = 800 °C	61.8	5.0	Chai et al.^166^
				*T*_R_ = 700 °C			
				steam = 5 mL h^–1^			
10Ni/Al_2_O_3_	PS-pine sawdust (5:5)	cp	fixed/fixed (0.5 g)	*T*_P_ = 800 °C	60.4	4.9	Chai et al.^166^
				*T*_R_ = 700 °C			
				steam = 5 mL h^–1^			

a wi, Wetness impregnation; cp, co-precipitation.

b *T*_P_, Pyrolysis temperature; *T*_R_, reforming temperature.

As observed, Arregi et al.^57^ analyzed the continuous pyrolysis and in-line catalytic reforming of pine wood waste and HDPE mixtures on a commercial Ni/Al_2_O_3_ catalyst (G90-LDP). The pyrolysis step was conducted in a CSBR at 500 °C, whereas the reforming one was performed in a fluidized bed reactor at a reforming temperature of 700 °C. In the mentioned study, the influence biomass/plastic (B/P) feeding ratio (25, 50, and 75 wt % HDPE) has on the product yields and catalyst deactivation was evaluated. As a result, these authors reported a marked attenuation of catalyst deactivation as well as a linear improvement of H_2_ production when HDPE feed was increased (from 17.5 wt % when a B/P weight ratio of 75/25 was used to 31.4 wt % when a B/P ratio of 25/75 was used). They also reported significant differences in the coke structure and nature (analyzed by TEM images), which were ascribed to the different composition of the volatiles fed into the reforming step. Moreover, a two-stage fixed bed reactor was used by Alvarez et al.^147^ in the co-pyrolysis-reforming of pine wood sawdust and different plastics (HDPE, PP, PS, and a real plastic mixture (RP)), with a biomass/plastic weight ratio of 80/20. The highest H_2_ production was observed when PP was co-fed with biomass (5.5 wt %), followed by HDPE in the feed (5.1 wt %).

More recently, Xu et al.^148^ analyzed the influence biomass/plastic ratio (in this case, rice husk (RH) and PE were selected as feedstock) has on the quality of the gaseous products in the catalytic steam reforming of the volatiles derived from co-pyrolysis of biomass and plastics. They reported a synergistic effect on gas and tar yields when PE was co-fed with rice husk, especially in the runs conducted using a RH/PE ratio of 50:50, wherein the H_2_ production reached 4.4 wt %.

### 4.2 Ni Supported Catalysts

The support significantly influences the catalyst activity and stability during the reaction. Thus, high specific surface area, adequate pore distribution and mechanical strength, and good thermal stability are required in order to obtain a suitable catalyst performance. Moreover, the acidity/basicity of the support may promote/hinder the deactivation by carbon deposition. Accordingly, metal oxide supports have been extensively analyzed in the steam reforming of oxygenates, using the aqueous fraction of bio-oil^107,167^ or model compounds^145,168^ as feedstocks. Similarly, studies have been conducted in a two-step process of biomass pyrolysis-steam reforming and they are summarized in Table 7. Hence, Miyazawa et al.^89^ carried out an activity test of different Ni supported catalysts (Ni/Al_2_O_3_, Ni/ZrO_2_, Ni/TiO_2_, Ni/CeO_2_ and Ni/MgO) in the steam reforming of the tar derived from the pyrolysis of cedar wood and observed that tar conversion at 650 °C decreased as follows: Ni/Al_2_O_3_ > Ni/ZrO_2_ > Ni/TiO_2_ > Ni/CeO_2_ > Ni/MgO. Besides, they concluded that the support only has influence on Ni dispersion, whereas Ni metal is responsible for tar conversion.

**Table 7:** Ni/Metal Oxide Supported Catalysts Reported in the Literature for the Pyrolysis and In-line Steam Reforming of Biomass and Plastic Wastes

Catalyst	Feed	Preparation method^a^	Reactor configuration	Operating conditions^b^	H_2_ conc. (vol %)	H_2_ prod. (wt %)	Ref.
20Ni/SiO_2_	wood pellets	iwi	screw-kiln/fixed (4 g min^–1^)	*T*_P_ = 500 °C	38.7	2.0	Efika et al.^102^
				*T*_R_ = 760 °C			
20Ni/SiO_2_	wood pellets	sg	screw-kiln/fixed (4 g min^–1^)	*T*_P_ = 500 °C	37.6	2.0	Efika et al.^102^
				*T*_R_ = 760 °C			
10Ni/SiO_2_	pine wood sawdust	wi	spouted/fluidized (0.75 g min^–1^)	*T*_P_ = 500 °C	55.7	1.6	Santamaria et al.^51,100^
				*T*_R_ = 600 °C			
				S/C = 7.7			
12Ni/MgO	cedar wood	iwi	dual fixed bed (0.06 g min^–1^)	*T* = 550–650 °C	32.4	2.3	Miyazawa et al.^89^
				S/C = 0.5			
6Ni/MgO	cotton stalk	commercial	bubbling fluidized/entrained flow/fixed (3.3 g min^–1^)	*T*_P_ = 600 °C	38.0	6.5	Chen et al.^170^
				*T*_G_ = 800 °C			
				*T*_R_ = 850 °C			
				S/B = 1–4			
6Ni/MgO	timber wood sawdust	commercial	bubbling fluidized/entrained flow/fixed	*T*_P_ = 600 °C	51.0	7.6	Ma et al.^169^
				*T*_G_ = 700–850 °C			
				*T*_R_ = 700–850 °C			
				S/B = 3			
10Ni/MgO	pine wood sawdust	wi	spouted/fluidized (0.75 g min^–1^)	*T*_P_ = 500 °C	61.6	9.1	Santamaria et al.^51,100^
				*T*_R_ = 600 °C			
				S/C = 7.7			
12Ni/TiO_2_	cedar wood	iwi	dual fixed bed (0.06 g min^–1^)	*T* = 550–650 °C	57.4	8.2	Miyazawa et al.^89^
				S/C = 0.5			
10Ni/TiO_2_	pine wood sawdust	wi	spouted/fluidized (0.75 g min^–1^)	*T*_P_ = 500 °C	57.9	7.2	Santamaria et al.^51,100^
				*T*_R_ = 600 °C			
				S/C = 7.7			
12Ni/ZrO_2_	cedar wood	iwi	dual fixed bed (0.06 g min^–1^)	*T* = 550–650 °C	52.7	6.6	Miyazawa et al.^89^
				S/C = 0.5			
10Ni/ZrO_2_	pine wood sawdust	wi	spouted/fluidized (0.75 g min^–1^)	*T*_P_ = 500 °C	65.4	10.7	Santamaria et al.^40,51,100^
				*T*_R_ = 600 °C			
				S/C = 7.7			
12Ni/CeO_2_	cedar wood	iwi	dual fixed bed (0.06 g min^–1^)	*T* = 550–650 °C	51.9	5.9	Miyazawa et al.^89^
				S/C = 0.5			
10Ni/MgO	PP	wi	fixed/fixed (1 g)	*T*_P_ = 500 °C	32.6	3.0	Wu and Williams^164^
				*T*_R_ = 800 °C			
				steam = 4.74 g h^–1^			
10Ni/MgO	LDPE	wi	fixed/fixed (2 g)	*T*_P_ = 500 °C	48.8	7.4	Huang et al.^171^
				*T*_R_ = 800 °C			
				S/P = 2			
10Ni/CeO_2_	PP	wi	fixed/fixed (1 g)	*T*_P_ = 500 °C	75.5	11.6	Wu and Williams^164^
				*T*_R_ = 800 °C			
				steam = 4.74 g h^–1^			
10Ni/CeO_2_	LDPE	wi	fixed/fixed (2 g)	*T*_P_ = 500 °C	57.2	12.2	Huang et al.^171^
				*T*_R_ = 800 °C			
				S/P = 2			
20Ni/ZrO_2_	PS	cp	fixed/fixed (0.3 g)	*T*_P_ = 500 °C	58.0	10.0	Zhou et al.^172^
				*T*_R_ = 500 °C			
				steam = 0.02 mL min^1^			
10Ni/Y_2_O_3_	LDPE	wi	fixed/fixed (2 g)	*T*_P_ = 500 °C	53.1	9.8	Huang et al.^171^
				*T*_R_ = 800 °C			
				S/P = 2			

a iwi, Incipient wetness impregnation; wi, wet impregnation; cp, co-precipitation; sg, sol–gel.

b *T*_P_ = Pyrolysis temperature; *T*_R_ = reforming temperature; *T*_G_ = gasification temperature.

Efika et al.^102^ investigated the production of synthesis gas in a two-step continuous screw-kiln reactor using wood pellets as a biomass feedstock. Thus, they analyzed the influence of the synthesis method for a Ni/SiO_2_ catalyst, which was prepared by incipient wetness impregnation and by a sol–gel method. The results showed that the catalyst prepared by the sol–gel method had a higher specific surface area (765 m^2^ g^–1^) and so led to a higher gas yield (54 wt %), whereas the gas yield obtained on the Ni/SiO_2_ catalyst prepared by the incipient wetness method was lower (49.8 wt %), which is associated with its lower specific surface area of 136 m^2^ g^–1^. The influence the support has on the activity and stability of Ni catalysts used in the reforming of pinewood sawdust fast pyrolysis volatiles was assessed by Santamaria et al.,^51,100^ with the selected supports being as follows: Al_2_O_3_, SiO_2_, MgO, TiO_2_ and ZrO_2_. They observed that despite Ni/Al_2_O_3_, Ni/ZrO_2_, and Ni/MgO catalysts were overall more active and stable over time on stream, the Ni/Al_2_O_3_ catalyst led to a remarkable coke deposition. However, a lower deactivation rate was evidenced for Ni supported on MgO and ZrO_2_ due to the properties conferred by these supports upon the catalysts, as are basicity and capacity for gasifying the coke precursors, respectively. The highest H_2_ production was achieved with Ni/ZrO_2_ catalyst (10.7 wt %), followed by Ni/Al_2_O_3_ (10.2 wt %) and Ni/MgO (9.1 wt %).

Moreover, Ma et al.^169^ investigated H_2_ production in a novel process integrating biomass pyrolysis, gas–solid simultaneous gasification, and the catalytic reforming process. Timber wood sawdust was the biomass selected, and a commercial Ni/MgO catalyst was used. H_2_ production significantly increased from 4.4 (using the two-step pyrolysis-catalytic reforming process) to 7.6 wt %. Likewise, Chen et al.^170^ evaluated the production of H_2_-rich gas from cotton stalks on a commercial Ni/MgO catalyst in the mentioned integrated process, and H_2_ production increased from 3.9 to 6.5 wt %, since the condensable gas and char is more efficiently used through simultaneous conversion.

Although the use of metal oxides supports have been scarcely investigated in the literature dealing with pyrolysis-reforming of plastics, details about the main studies have been summarized in Table 7. Thus, the suitability of the two-step pyrolysis-reforming process for H_2_ production from polypropylene (PP) was analyzed by Wu and Williams,^164^ who synthesized several nickel based catalysts. Among them, different metal oxides were selected: Al_2_O_3_, MgO, and CeO_2_. However, these Ni supported catalysts showed lower activity for H_2_ production compared to other supported or promoted catalysts tested (Ni/ZSM-5, Ni/CeO_2_/Al_2_O_3_ and NiMgAl).

Recently, several Ni supported catalysts were analyzed in the steam reforming of LDPE pyrolysis volatiles for syngas production by Huang et al.^171^ In this study, the great influence the support selected has on catalytic activity, selectivity, and coke formation was evidenced, with Ni/CeO_2_ catalyst being the most active, followed by Al_2_O_3_ supported catalysts and Ni/Y_2_O_3_.

Furthermore, the use of alternative supports, such as dolomite, char, or active carbon, are gaining increasing attention due to their lower cost. Moreover, the concentration of alkaline metals, such as K and Ca, in the biochar produced from biomass pyrolysis may promote the decomposition of hydrocarbons and the water gas shift reaction during the reforming process,^173^ making this material suitable as catalyst support. The suitable properties of biochar and coal char, i.e., high specific surface area, optimized pore volume, excellent thermal stability, and abundant surface functional groups, have promoted their use in the reforming of biomass tar. Recently, Ren et al.^174^ discussed in detail the preparation, modification, and characteristics of biochar and coal char as well as their application in biomass tar reforming.

Table 8 compares the studies reported in the literature for pyrolysis and in-line reforming of biomass. Thus, some studies have been conducted with continuous biomass feed using a fluidized or entrained flow reactor in the pyrolysis step and a fixed bed reactor in the reforming one. Thus, Xiao et al.^61^ compared a commercial Ni/Al_2_O_3_ and a Ni/BCC (brown coal char) catalyst in the continuous pyrolysis-reforming of two different feedstocks (wood chips and pig manure compost). They obtained lower tar content, higher coking resistance, and higher H_2_ production (9.3 wt % daf) when Ni/BCC was used. Liu et al.^160^ investigated the syngas production in the catalytic reforming of pyrolysis volatiles from pine sawdust with a continuous feed of 4 g min^–1^. Five innovative slag carriers were selected as catalytic supports ((magnesium slag (MS), steel slag (SS), blast furnace slag (BFS), pyrite cinder (PyC), and calcium silicate slag (CSS)) and Ni as metal active phase. Under the operating conditions selected (pyrolysis temperature of 900 °C and reforming one of 800 °C), the H_2_ production decreased as follows: Ni/MS > Ni/γ-Al_2_O_3_ > Ni/SS > Ni/BFS > Ni/CSS > Ni/PbyC.

**Table 8:** Other Ni/Supported Catalysts Reported in the Literature for Biomass Pyrolysis and In-line Steam Reforming

Catalyst	Feed	Preparation method^b^	Reactor configuration	Operating conditions^d^	H_2_ conc. (vol %)	H_2_ prod. (wt %)	Ref.
19.2Ni/BCC^a^	red pine wood	i.e	fluidized/fixed (1–2 g min^–1^)	*T*_P_ = 530–700 °C	60.0	9.3^c^	Xiao et al.^61^
				*T*_R_ = 550–710 °C			
19.2Ni/BCC^a^	PC^a^	i.e	fluidized/fixed (1–2 g min^–1^)	*T*_P_ = 530–700 °C	43.9	5.0^c^	Xiao et al.^61^
				*T*_R_ = 550–710 °C			
3Ni/MS^a^	pine sawdust	wi	entrained-flow reactor/fixed (4 g min^–1^)	T_P_ = 900 °C	52.6	6.9	Liu et al.^160^
				T_R_ = 800 °C			
3Ni/SS^a^	pine sawdust	wi	entrained-flow reactor/fixed (4 g min^–1^)	*T*_P_ = 900 °C	41.5	4.4	Liu et al.^160^
				*T*_R_ = 800 °C			
3Ni/BFS^a^	pine sawdust	wi	entrained-flow reactor/fixed (4 g min^–1^)	*T*_P_ = 900 °C	41.4	4.2	Liu et al.^160^
				*T*_R_ = 800 °C			
0.88Ni/Char	mallee	i.e	fluidized/fixed (0.1 g min^–1^)	*T*_R_ = 500–850 °C			Min et al.^182^
15.6Ni/HSL^a^	corncob	i.e	fixed/fixed (1 g)	*T*_P_ = 900 °C	70.0	10.0^c^	Ren et al.^175^
				*T*_R_ = 500–700 °C			
20Ni/CD^a^	Japanese cypress	wi	fixed/fixed (1 g)	*T* = 450 °C	35.9		Kannari et al.^159^
20Ni/CDA^a^	Japanese cypress	wi	fixed/fixed (1 g)	*T* = 450 °C	38.7	5.8^c^	Kannari et al.^159^
10Ni/dolomite	rice husk	wi	fixed/fixed (4 g)	*T*_P_ = 950 °C	65.2	6.1	Waheed et al.^179^
				*T*_R_ = 850–1050 °C			
				S/B = 0.46–2.28			
10Ni/dolomite	rice husk	wi	fixed/fixed (4 g)	*T*_P_ = 950 °C	59.1	5.1	Waheed and Williams^180^
				*T*_R_ = 950 °C			
				S/B = 1.37			
10Ni/dolomite	sugar cane bagasse	wi	fixed/fixed (4 g)	*T*_P_ = 950 °C	57.0	5.1	Waheed and Williams^180^
				*T*_R_ = 950 °C			
				S/B = 1.37			
10Ni/dolomite	wheat straw	wi	fixed/fixed (4 g)	*T*_P_ = 950 °C	58.2	4.9	Waheed and Williams^180^
				*T*_R_ = 950 °C			
				S/B = 1.37			
15Ni/WC^a^	wheat straw	wi	fixed/fixed (1 g)	*T*_P_ = 500 °C	62.0	4.2	Yao et al.^173^
				*T*_R_ = 600–900 °C			
15Ni/RC^a^	wheat straw	wi	fixed/fixed (1 g)	*T*_P_ = 500 °C	48.0	3.0	Yao et al.^173^
				*T*_R_ = 600–900 °C			
15Ni/CC^a^	wheat straw	wi	fixed/fixed (1 g)	*T*_P_ = 500 °C	64.0	9.2	Yao et al.^173^
				*T*_R_ = 600–900 °C			
5-20Ni/AC^a^	wheat straw	wi	fixed/fixed (1 g)	*T*_P_ = 500 °C	50.0	4.0	Yao et al.^173^
				*T*_R_ = 600–900 °C			
2.5Ni/CS^a^	apple branch	wi	fixed/fixed (0.6 g)	*T* = 650 °C	55.0	3.9^c^	Guan et al.^183^
0.5Ni/zeolite	seaweed	iwi	fixed/fixed (0.6 g)	*T* = 510–660 °C	52.5	4.9	Kaewpanha et al.^184^
Ni/RHA^a^	rice husk	iwi	fixed/fixed (10 g)	*T*_P_ = 800 °C	28.8	5.3	Shen et al.^185^
				*T*_R_ = 600–900 °C			
Ni/Hidrochar (0.1–1 M)	sewage sludge	HTC	fixed/fixed (1 g)	*T*_P_ = 600 °C	62.0	10.9	Gai et al.^181^
				*T*_R_ = 500–900 °C			
				GHSV = 3700 h^–1^ (2nd stage)			

a BCC, Brown coal char; MS, magnesium slag; SS, steel slag; BFS, blast furnace slag; HSL, HCl treatment Shengli lignite; CD, chicken dropping; CDA, chicken dropping ash; WC, biochar from wheat straw; RC, biochar from rice husk; CC, biochar from cotton stalk; AC, active carbon; CS, calcined scallop shell; RHA, rice husk ash.

b iwi, Incipient wetness impregnation; wi, wet impregnation; ie, ion- exchange; HTC, hydrothermal carbonization.

c H_2_ production defined as g_H2_/100 g_biomass, daf_ (dry and ash free).

d T_P_ = Pyrolysis temperature; *T*_R_ = reforming temperature.

Nevertheless, most of these studies have been performed in batch operation using a two-step fixed bed reactor. Thus, Kannari et al.^159^ used chicken droppings (CD) and chicken dropping ash (CDA) as catalytic support on a Ni based catalyst for decomposing tar derived from Japanese cypress pyrolysis. The results revealed that Ni/CDA leads to higher H_2_ production than the commercial Ni/Al_2_O_3_ catalyst (5.8 wt % vs 5.0 wt % daf) and a lower amount of carbon deposition. Ren et al.^175^ synthesized a layered Ni/modified lignite char (Ni/HSL) and analyzed its performance in a two stage fixed bed reactor using corncob as the raw material. They reported optimum activity of Ni/HSL compared to Ni/Al_2_O_3_, with a H_2_ production of 10.0 wt % daf. The suitability of this lignite char support was also evident in previous studies conducted by this research group.^176−178^

Moreover, Waheed et al.^179^ investigated the conditions for H_2_ production from rice husk in a two-step pyrolysis/catalytic reforming process containing Ni supported on dolomite catalyst. The highest H_2_ production was obtained when the temperature was increased to 1050 °C in the reforming step (6.1 wt %). Besides, different biomasses (rice husk, sugar cane bagasse, and wheat straw) were analyzed by these authors^180^ on a 10 wt % Ni/dolomite catalyst, obtaining the highest H_2_ production when rice husk was used as the feedstock (5.1 wt %).

The use of biochar as catalyst support is gaining increasing interest in the literature. Yao et al.^173^ used three types of biochar (obtained from the pyrolysis of wheat straw (WC), rice husk (RC), and cotton stalk (CC)) as a support of a 15 wt % Ni catalyst in the pyrolysis/reforming of wheat straw. They concluded that biochar is a promising catalytic support for this process, with the cotton-char supported Ni leading to the best results in terms of H_2_ concentration and production (64.0 vol % and 9.2 wt %, respectively). Moreover, Gai et al.^181^ developed a mild one-step hydrothermal synthesis method for the production of well-dispersed metallic nickel nanoparticles on hydrothermal carbons derived from waste biomass (hydrochar). Under the optimum synthesis conditions (calcination temperature of 700 °C), this catalyst led to attenuation of coke deposition and resistance of nickel agglomeration during the catalytic reforming process. Thus, the formation of hydrogen-rich syngas was promoted, obtaining a H_2_ production of 10.9 wt %.

**Figure 13:**
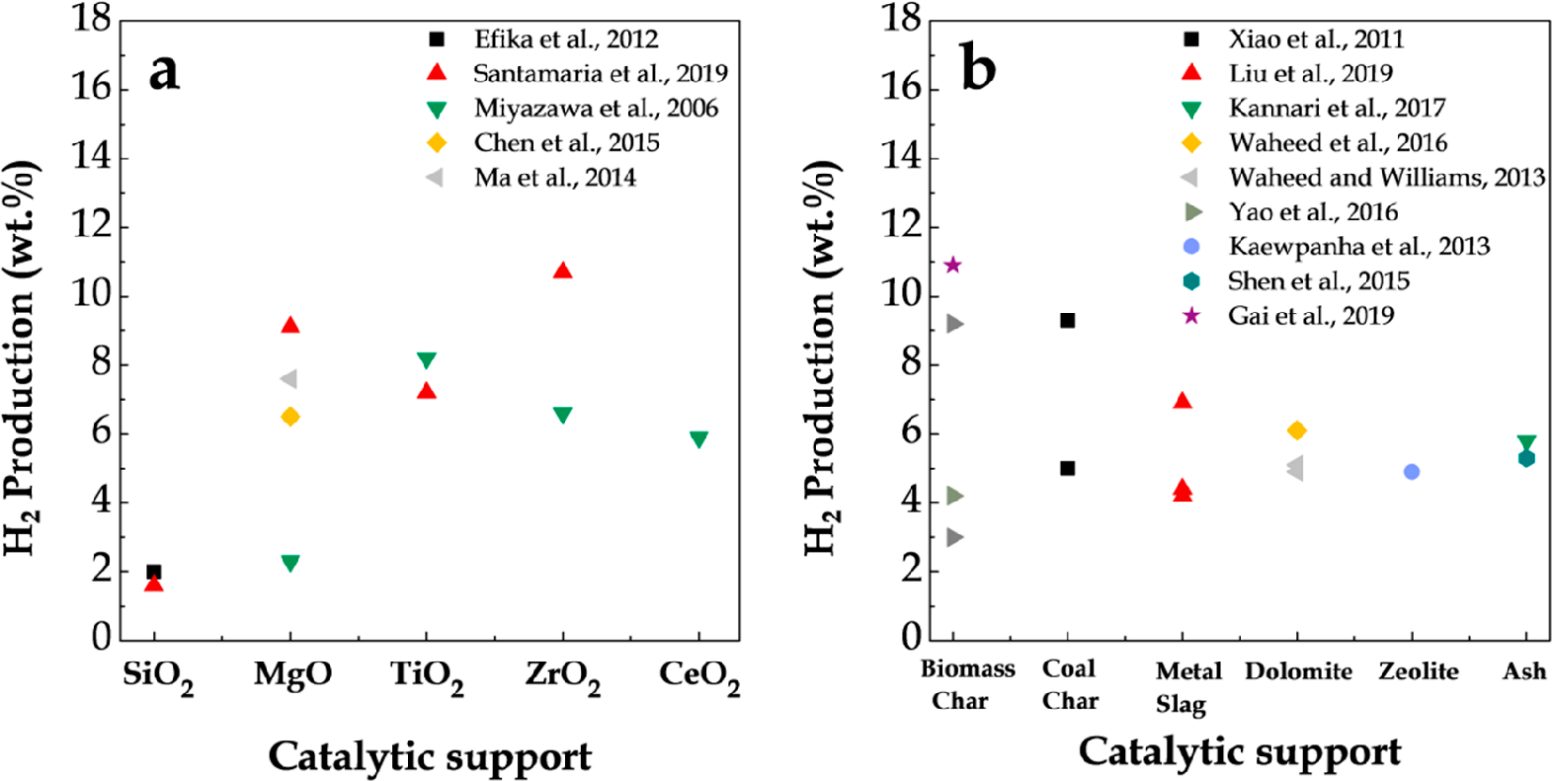
Influence of the support on hydrogen production in the in-line biomass pyrolysis-reforming: Metal oxide supports (a), and other catalytic supports (b). Efika et al., 2012;^102^ Santamaria et al., 2019;^100^ Miyazawa et al., 2006;^89^ Chen et al., 2015;^170^ Ma et al., 2014;^169^ Xiao et al., 2011;^61^ Liu et al., 2019;^160^ Kannari et al., 2017;^159^ Waheed et al., 2016;^179^ Waheed and Williams, 2013;^180^ Yao et al., 2016;^173^ Kaewpanha et al., 2013;^184^ Shen et al., 2015;^185^ Gai et al., 2019.^181^

Figure 13 compares the main supports used in the steam reforming of biomass pyrolysis volatiles. Among the different supports selected, metal oxides alternative to Al_2_O_3_ have been extensively analyzed (Figure 13a) in order to attenuate the fast catalyst deactivation by coke deposition promoted by the acid properties of the Al_2_O_3_ support. In this regard, supports with basic properties, namely SiO_2_, MgO, ZrO_2_, or CeO_2_ are known to delay coke formation.^100^ It is to note that the results presented in Figure 13 only account for their initial performance, and catalyst stability should therefore be monitored throughout the reaction for the selection of the more suitable catalyst. However, this aspect has been scarcely studied in the literature.

As mentioned before, the metal dispersion on the catalytic support plays a key role in the initial catalyst activity. Besides, other factors such as adequate metal–support interactions, catalyst reducibility, mechanical strength and thermal stability greatly influence the performance of the reforming catalyst.

As observed in Figure 13a, supported catalysts are in general active for the reforming of the biomass pyrolysis volatiles. In the case of SiO_2_, although this support has high specific surface area and allows for a high Ni dispersion, its fine porous structure hinders the accessibility of oxygenate bulky molecules, leading to a considerable reduction in activity.^51^ The good performance of a commercial MgO supported catalyst was evidenced in a three-step process (pyrolysis-gasification-reforming) by Ma et al.^169^ and Chen et al.,^170^ as they obtained H_2_ productions in the 6.5–7.6 wt % range. Santamaria et al.^100^ reported that, in spite of the poor porous structure of the MgO support (specific surface area of 1 m^2^ g^–1^) and its low reducibility (strong metal support-interaction), a suitable activity and stability was observed (with a H_2_ production of 9.0 wt %) due to the external location of Ni particles, which enhanced the accessibility of the bulky oxygenate molecules. Nevertheless, Miyazawa et al.,^89^ ascribed the poor activity of the MgO supported catalyst to the low metal dispersion obtained when this catalyst was tested.

The suitable features of ZrO_2_ (redox properties, mechanical strength and thermal stability) and TiO_2_ supports (good reducibility and attenuation of coke formation) have promoted their use in the pyrolysis-reforming strategy.

Figure 13b shows the main biomass pyrolysis-reforming studies for other supports, such as biomass derived char, coal derived char, metal slag, dolomite, zeolite or biomass derived ashes. The use of these alternative supports is motivated by their lower cost compared to the conventional metal oxide supports. As regards the biochar derived supports, the presence of alkaline metals make these materials suitable for this purpose.^173^ Moreover, the high surface provided by these supports ensures suitable metal dispersion. Nevertheless, these supported catalysts are not suitable for reaction-regeneration cycles due to their limited stability under oxygen atmosphere, which hinders their use in large-scale units.

As regards the use of metal slag as catalytic support, it provides the additional advantage of recycling an industrial waste residue. Besides, the metal additives present in the Ni-based slag catalysts resulted in a better Ni dispersion and promoted synergistic catalysis effects, which resulted in an enhancement of catalyst activity and coke deposition resistance.^160^

However, to provide reliable conclusions concerning the suitability of each support, further research must be conducted in terms of catalyst activity, stability, and regeneration. Generally, the use of alternative low-cost supports (Figure 13b) leads to lower H_2_ productions compared to metal oxide supports. Among these latter ones, ZrO_2_ is regarded as a promising catalyst support for use in reaction-regeneration cycles.^100^ Besides, as mentioned before, any comparison of the studies showed in Figure 13 should consider the catalyst design (synthesis method and conditions), the operating parameters used in the runs and the type of biomass used as feedstock.

The use of alternative supports in the pyrolysis-reforming of plastic wastes has been analyzed in the literature, and details about the main studies are shown Table 9. In these studies, zeolite supports have been preferably selected for the production of H_2_ or syngas. Yao et al.^186^ analyzed different zeolite supported nickel catalysts (Ni/ZSM5–30, Ni/β-zeolite-25 and the Ni/Y-zeolite-30 catalysts) in a two-step fixed bed unit wherein HDPE was pyrolyzed at 500 °C and the volatiles produced were reformed at a temperature range of 650–850 °C. At the highest catalytic temperature and using a steam feeding rate of 6 g h^–1^, the Ni/ZSM5–30 catalyst revealed the best performance, with a H_2_ production of 13.2 wt %. Moreover, the Ni/Y-zeolite shows the worst performance in terms of syngas production and highest coke formation, which was ascribed to the ultramicropores in this zeolite support. In a similar experimental unit, but using PP as feedstock, Wu and Williams^164^ reported the effectiveness of Ni/ZSM-5 catalyst for the production of hydrogen (19.0 wt %), as mainly filamentous coke was deposited on this catalyst, which has little influence on the catalytic activity.

**Table 9:** Other Ni/Supported Catalysts Reported in the Literature for the Pyrolysis and In-line Steam Reforming of Plastic Wastes

Catalyst	Feed	Preparation method^a^	Reactor configuration	Operating conditions^b^	H_2_ conc. (vol %)	H_2_ prod. (wt %)	Ref.
10Ni/Y-30	HDPE	wi	fixed/fixed (1 g)	*T*_P_ = 500 °C	53.6	11.6	Yao et al.^186^
				*T*_R_ = 650–850 °C			
				steam = 0–6 g h^–1^			
10Ni/β-zeolite-25	HDPE	wi	fixed/fixed (1 g)	*T*_P_ = 500 °C	55.8	12.3	Yao et al.^186^
				*T*_R_ = 650–850 °C			
				steam = 0–6 g h^–1^			
10Ni/ZSM5-30 (Si/Al ratio; 30)	HDPE	wi	fixed/fixed (1 g)	*T*_P_ = 500 °C	56.2	13.2	Yao et al.^186^
				*T*_R_ = 650–850 °C			
				steam = 0–6 g h^–1^			
10Ni/ZSM5-50 (Si/Al ratio; 50)	HDPE	wi	fixed/fixed (1 g)	*T*_P_ = 500 °C	57.0	12.1	Yao et al.^186^
				*T*_R_ = 650–850 °C			
				steam = 0–6 g h^–1^			
10Ni/ZSM5-80 (Si/Al ratio; 80)	HDPE	wi	fixed/fixed (1 g)	*T*_P_ = 500 °C	56.2	12.0	Yao et al.^186^
				*T*_R_ = 650–850 °C			
				steam = 0–6 g h^–1^			
10Ni/ZSM-5	PP	wi	fixed/fixed (1 g)	*T*_P_ = 500 °C	63.6	19.0	Wu and Williams^164^
				*T*_R_ = 800 °C			
				steam = 4.74 g h^–1^			
10Ni/ZSM-5	LDPE	wi	fixed/fixed (2 g)	*T*_P_ = 500 °C	45.9	5.6	Huang et al.^171^
				*T*_R_ = 800 °C			
				S/P = 2			

a wi, Wet impregnation.

b *T*_P_ = Pyrolysis temperature; *T*_R_ = reforming temperature.

### 4.3 Promoter Incorporation into Ni-Supported Catalysts

The incorporation of a promoter contributes to improving the activity, selectivity, and stability, as it modifies the active phase and/or the support. Thus, the promoter can positively enhance the thermal stability, mechanical strength, reducibility, metal dispersion, and coke resistance.

The elements that may act as promoters may be grouped as follows: (i) alkali metals, such as Li, Na, K, Rb, or Cs, which modify the reducibility of the metal active phase and enhance the initial catalyst activity;^187^ (ii) alkaline-earth metals, such as Mg, Ca, Sr, and Ba, which reduce the acidity of the catalyst and enhance water adsorption and OH surface mobility, thereby reducing coke deposition rate;^188^ (iii) rare earth oxides, such as La_2_O_3_, CeO_2_, or Pr_6_O_11_, which modify the metal–support interaction, improve the dispersion of the active phase, provide redox properties, reduce metal sintering, and so hinder carbon deposition;^117,189,190^ and (iv) transition metal oxides, such as ZrO_2_, MnO_*x*_ or ZnO, among others, which influence the metal–support interaction, decrease the acidity of the catalyst and enhance the coke deposition resistance.^88,117^

Several authors have considered the addition of secondary metals (transition metals, such as Fe, Co, Mn, or Cu, and noble metals, such as Ru, Rh, Pd, or Pt) as promoters to form bimetallic catalysts. These types of catalysts are described in the following section (section 4.4).

Table 10 summarizes the main researches reported in the literature on Ni-promoted Al_2_O_3_ catalyst for biomass pyrolysis and in-line steam reforming. Thus, CeO_2_ is regarded as a promising promoter for reforming catalysts due to its redox characteristics and oxygen storage capacity. Accordingly, Kimura et al.^124^ prepared two Ni/CeO_2_-Al_2_O_3_ catalysts by co-impregnation and sequential impregnation, respectively, obtaining better catalytic performance in terms of tar conversion and coke deposition with the catalyst prepared by co-impregnation, as this synthesis method leads to a stronger interaction between Ni and CeO_2_. Efika et al.^102^ compared different Ni based catalysts, obtaining the lowest H_2_ concentration and production (43.1% and 2.2 wt %, respectively) with a Ni/CeO_2_-Al_2_O_3_ one containing a high CeO_2_ load (20 wt %). Furthermore, the enhancement of the Ni/Al_2_O_3_ catalyst performance by the addition of different promoters (La_2_O_3_, CeO_2_, and MgO) in the continuous biomass pyrolysis-reforming was analyzed by Santamaria et al.^191,192^ In these studies, all the promoted catalysts revealed a similar initial activity, with the highest H_2_ production by mass unit being for the Ni/CeO_2_-Al_2_O_3_ catalyst (11.5 wt %), followed by Ni/La_2_O_3_-Al_2_O_3_ (10.0 wt %) and Ni/MgO-Al_2_O_3_ (9.7 wt %) catalysts. The stability was greatly improved by the incorporation of CeO_2_ and La_2_O_3_ promoters due to the characteristic features conferred upon the catalysts. Thus, CeO_2_ provides redox properties, high oxygen storage capacity, and capability to favor water adsorption, whereas the basicity and water adsorption capability of La_2_O_3_ promoter inhibits the formation of coke and leads to the gasification of the coke deposited. Despite the low reducibility of Ni/MgO-Al_2_O_3_ due to MgAl_2_O_4_ spinel phase formation, which leads to a poorer stability of this catalysts compared to Ni/Al_2_O_3_, these authors greatly improved the performance of MgO promoted catalyst by the modification of the calcination temperature in the synthesis step.^193^

**Table 10:** Promoted Ni–Al_2_O_3_ Catalysts Reported in the Literature for the Pyrolysis and In-line Steam Reforming of Biomass

Catalyst	Feed	Preparation method^a^	Reactor configuration	Operating conditions^c^	H_2_ conc. (vol %)	H_2_ prod. (wt %)	Ref.
20Ni/CeO_2_-Al_2_O_3_	wood pellets	iwi	screw-kiln/fixed (4 g min^–1^)	*T*_P_ = 500 °C	43.1	2.2	Efika et al.^102^
				*T*_R_ = 760 °C			
12Ni/CeO_2_-Al_2_O_3_	cedar wood	swi cwi	dual fixed (0.06 g min^–1^)	*T* = 550 °C	57.5	6.9	Kimura et al.^124^
10Ni/CeO_2_-Al_2_O_3_	pine wood sawdust	wi	spouted/fluidized (0.75 g min^–1^)	*T*_P_ = 500 °C	64.7	10.5	Santamaria et al.^192^
				*T*_R_ = 600 °C			
				S/C = 7.7			
10Ni/La_2_O_3_-Al_2_O_3_	pine wood sawdust	wi	spouted/fluidized (0.75 g min^–1^)	*T*_P_ = 500 °C	63.6	10.0	Santamaria et al.^191^
				*T*_R_ = 600 °C			
				S/C = 7.7			
12Ni/MnO_*x*_-Al_2_O_3_	cedar wood	cp	dual fixed (0.06 g min^–1^)	*T* = 550–650 °C	46.5	5.0^b^	Koike et al.^88^
				S/C = 0.57			
10Ni/MgO-Al_2_O_3_	pine wood sawdust	wi	spouted/fluidized (0.75 g min^–1^)	*T*_P_ = 500 °C	63.0	9.7	Santamaria et al.^192^
				*T*_R_ = 600 °C			
				S/C = 7.7			
10Ni/MgO-Al_2_O_3_ (*T*_calc_ = 700/700)	pine wood sawdust	wi	spouted/fluidized (0.75 g min^–1^)	*T*_P_ = 500 °C	64.4	10.3	Santamaria et al.^193^
				*T*_R_ = 600 °C			
				S/C = 7.7			
12Ni-Mg-Al	cedar wood	cp	dual fixed (0.06 g min^–1^)	*T* = 550–650 °C	54.9	7.9	Li et al.^87^
				S/C = 0.38			
Ni-Mg-Al	cellulose	cp	fixed/fixed (0.5 g)	*T*_P_ = 500 °C	54.7	4.5	Wu et al.^195^
				*T*_R_ = 800 °C			
Ni-Mg-Al	lignin	cp	fixed/fixed (0.5 g)	*T*_P_ = 500 °C	55.1	2.8	Wu et al.^195^
				*T*_R_ = 800 °C			
Ni-Ca-Al	lignin	cp	fixed/fixed (0.5 g)	*T*_P_ = 500 °C	54.6	3.6	Wu et al.^195^
				*T*_R_ = 700–900 °C			
Ni/CaAlOx	wood sawdust	cp	fixed/fixed (0.5 g)	*T*_P_ = 500 °C	46.0	3.1	Chen et al.^82^
				*T*_R_ = 800 °C			
				steam = 0.05 g min^–1^			
9Ni/K_2_CO_3_–Al_2_O_3_	rice husk	wi	drop-tube fixed (120 mg)	*T* = 800 °C	33.3	1.3	Kuchonthara et al.^103^
5-35 NiZnAlOx	wood sawdust	cp	fixed/fixed (0.8 g)	*T*_P_ = 535 °C	48.1	4.0	Dong et al.^196^
				*T*_R_ = 800 °C			

a iwi, Incipient wetness impregnation; swi, sequential wetness impregnation; cwi, co-impregnation; wi, wet impregnation; cp, co-precipitation.

b H_2_ production defined as g_H2_/100 g_biomass, daf_ (dry and ash free).

c *T*_P_ = Pyrolysis temperature; *T*_R_ = reforming temperature.

The promotion of Ni/Al_2_O_3_ catalyst with MgO has also been analyzed by Li et al.^194^ in the pyrolysis-reforming of cedar wood. Thus, the influence the composition and reduction conditions have on the catalytic performance was determined, observing that the Ni/MgO-Al_2_O_3_ catalyst with Ni/Mg/Al weight ratio of 9/66/25 exhibited much higher activity, resistance to coke deposition, and stability than Ni based catalysts supported on Al_2_O_3_ and MgO. Likewise, Wu et al.^195^ analyzed the influence of the reforming temperature on the pyrolysis/reforming of lignin on a Ni-Ca-Al catalyst, obtaining an improvement in H_2_ production from 2.1 wt % at 700 °C to 3.6 wt % at 900 °C. Moreover, they tested a Ni-Mg-Al catalyst with three different biomass components (lignin, cellulose, and xylan) and reported the highest H_2_ production for the cellulose feedstock (4.5 wt %).

The influence of adding alkali metals (K_2_CO_3_) on a Ni/Al_2_O_3_ catalyst was analyzed by Kuchonthara et al.^103^ in the steam reforming of rice husk-derived tar. They obtained better results with the promoted Ni/K_2_CO_3_-Al_2_O_3_ catalyst in terms of carbon conversion and H_2_ production, attributing this fact to the effect of this promoter by attenuating the thermal sintering of Ni-catalysts.

Similarly, Koike et al.^88^ used a MnO_*x*_ promoted Ni/Al_2_O_3_ catalyst in the steam reforming of the volatiles from cedar wood pyrolysis and proved that an optimum catalyst composition, in which the interaction between Ni metal and MnO_*x*_ is positively modified, enhances catalytic activity, and minimizes coke deposition. However, an excess of MnO_*x*_ involves a decrease in the number of surface Ni atoms, reducing catalytic activity.

The influence of adding promoters to Ni/Al_2_O_3_ catalysts on the catalytic steam reforming of the volatiles from plastic waste pyrolysis has also been assessed in the literature, and the main studies are presented in Table 11. Thus, Arregi et al.^55^ evaluated the performance of Ni/Al_2_O_3_ and two promoted catalysts (Ni/CeO_2_-Al_2_O_3_ and Ni/La_2_O_3_-Al_2_O_3_) in a continuous bench scale pyrolysis-reforming plant using PP as the feedstock. The results evidenced a suitable performance of all the synthesized catalysts, with the best results in terms of conversion, hydrogen production, and coke deposition being obtained when the La_2_O_3_ promoted catalyst was tested (H_2_ production of 34.9 wt %).

**Table 11:** Promoted Ni-Al_2_O_3_ Catalysts Reported in the Literature for the Pyrolysis and In-line Steam Reforming of Plastic Wastes

Catalyst	Feed	Preparation method^a^	Reactor configuration	Operating conditions^b^	H_2_ conc. (vol %)	H_2_ prod. (wt %)	Ref.
10Ni/La_2_O_3_-Al_2_O_3_	PP	wi	spouted/fluidized (0.75 g min^–1^)	*T*_P_ = 500 °C	71.2	34.9	Arregi et al.^55^
				*T*_R_ = 700 °C			
				S/C = 3.1			
10Ni/CeO_2_-Al_2_O_3_	PP	wi	spouted/fluidized (0.75 g min^–1^)	*T*_P_ = 500 °C	71.1	33.7	Arregi et al.^55^
				*T*_R_ = 700 °C			
				S/C = 3.1			
10Ni/CeO_2_-Al_2_O_3_	PP	wi	fixed/fixed (1 g)	*T*_P_ = 500 °C	63.8	19.5	Wu and Williams^164^
				*T*_R_ = 800 °C			
				steam = 4.74 g h^–1^			
Ni-Ce-Al 1:1:1	PP	cp	fixed/fixed (2 g)	*T*_P_ = 500 °C	55.6	12.6	Nahil et al.^197^
				*T*_R_ = 800 °C			
				steam = 4.74 g h^–1^			
Ni-Mg-Al 1:1:2	PP	cp	fixed/fixed (1 g)	*T*_P_ = 500 °C	61.8	22.3	Wu and Williams^164^
				*T*_R_ = 800 °C			
				steam = 4.74 g h^–1^			
Ni-Mg-Al 1:1:1	PP	cp	fixed/fixed (1 g)	*T*_P_ = 500 °C	65.0	26.6	Wu and Williams^47^
				*T*_R_ = 800 °C			
				steam = 4.74 g h^–1^			
Ni-Mg-Al 1:1:1	PS	cp	fixed/fixed (1 g)	*T*_P_ = 500 °C	58.7	18.5	Wu and Williams^47^
				*T*_R_ = 800 °C			
				steam = 4.74 g h^–1^			
Ni-Mg-Al 1:1:1	HDPE	cp	fixed/fixed (1 g)	*T*_P_ = 500 °C	65.0	26.0	Wu and Williams^47^
				*T*_R_ = 800 °C			
				steam = 4.74 g h^–1^			
Ni-Mg-Al 1:1:1	PP: 26.9 wt %	cp	fixed/fixed (1 g)	*T*_P_ = 500 °C	66.3	25.3	Wu and Williams^47^
	PS: 16.8 wt %			*T*_R_ = 800 °C			
	HDPE: 56.3 wt %			steam = 4.74 g h^–1^			
Ni-Mg-Al 1:1:1	real-world plastics (RP)	cp	fixed/fixed (1 g)	*T*_P_ = 500 °C	67.5	23.6	Wu and Williams^47^
				*T*_R_ = 800 °C			
				steam = 4.74 g h^–1^			
Ni-Mn-Al 1:1:1	PP	cp	fixed/fixed (2 g)	*T*_P_ = 500 °C	62.7	14.3	Nahil et al.^197^
				*T*_R_ = 800 °C			
				steam = 4.74 g h^–1^			
Ni-Ca-Al 1:1:1	PP	cp	fixed/fixed (2 g)	*T*_P_ = 500 °C	58.3	13.7	Nahil et al.^197^
				*T*_R_ = 800 °C			
				steam = 4.74 g h^–1^			
Ni-Zn-Al 1:1:1	PP	cp	fixed/fixed (2 g)	*T*_P_ = 500 °C	52.7	9.2	Nahil et al.^197^
				*T*_R_ = 800 °C			
				steam = 4.74 g h^–1^			

a wi, Wet impregnation; cp, co-precipitation.

b *T*_P_ = Pyrolysis temperature; *T*_R_ = reforming temperature.

Moreover, Nahil et al.^197^ analyzed the influence of promoter incorporation into a nickel based catalyst for the co-production of hydrogen and carbon nanotubes in the pyrolysis–catalytic reforming of PP. The metal oxides selected as promoters were Zn, Mg, Ca, Ce, and Mn, and all the catalysts were prepared by the co-precipitation method. The Ni-Mn-Al was reported as the optimum catalyst, with H_2_ production being 14.3 wt %.

Besides, a wide range of studies have been performed using Mg as the promoter on Ni/Al_2_O_3_ catalysts.^47,150,164^ Namely, the production of H_2_ in a two-step fixed bed reactor was investigated by Wu and Williams,^47^ who analyzed the influence the plastic type (PP, PS, HDPE, their mixtures, and real-world plastics) has on the product distribution. The highest H_2_ production was obtained when PP was fed (26.6 wt %), followed by HDPE (26.0 wt %), whereas PS presented the lowest one (18.5 wt %). The use of real-world plastics (waste plastic processing) allowed a H_2_ production of 23.6 wt %.

It is to note that, although increasing attention has been paid in recent years to the joint valorization of biomass and plastic mixtures by the two-step pyrolysis-reforming process, the studies performed are still scarce. Table 12 shows the main studies reported in the literature wherein promoted Ni based catalysts were selected for the pyrolysis and in-line steam reforming of biomass and plastic waste mixtures. Thus, Kumagai et al.^198^ prepared a Ni-Mg-Al catalyst by the co-precipitation method and analyzed the influence Ca loading and catalyst calcination temperature have on the production of H_2_ in pyrolysis–reforming runs using a mixture of wood sawdust and PP. The highest hydrogen production (6.2 wt %) was obtained in the presence of a Ca containing catalyst having a molar ratio of Ni/Mg/Al/Ca = 1:1:1:4, calcined at 500 °C.

**Table 12:** Promoted Ni Based Catalysts Reported in the Literature for the Pyrolysis and In-line Steam Reforming of Biomass and Plastic Mixtures

Catalyst	Feed	Preparation method^a^	Reactor configuration	Operating conditions^b^	H_2_ conc. (vol %)	H_2_ prod. (wt %)	Ref.
35.4Ni-Mg-Al 1:1:1	PP-wood sawdust	cp	fixed/fixed (2 g)	*T*_P_ = 600 °C	51.0	6.3	Kumagai et al.^198^
				*T*_R_ = 800 °C			
				steam = 0.05 g min^–1^			
21.1Ni-Mg-Ca-Al 1:1:1:2	PP-wood sawdust PP	cp	fixed/fixed (2 g)	*T*_P_ = 600 °C	51.5	6.2	Kumagai et al.^198^
				*T*_R_ = 800 °C			
				steam = 0.05 g min^–1^			
5-20Ni-Ca-C	LDPE-pine sawdust (5:5)	cp	fixed/fixed (0.5 g mixture)	*T*_P_ = 700 °C	86.7	23.1	Chai et al.^53^
				*T*_R_ = 600 °C			
				steam = 5 mL h^–1^			
10Ni-Ca-C	HDPE-pine sawdust (5:5)	cp	fixed/fixed (0.5 g mixture)	*T*_P_ = 800 °C	80.4	14.0	Chai et al.^166^
				*T*_R_ = 700 °C			
				steam = 5 mL h^–1^			
10Ni-Ca-C	PP-pine sawdust (5:5)	cp	fixed/fixed (0.5 g mixture)	*T*_P_ = 800 °C	59.4	13.8	Chai et al.^166^
				*T*_R_ = 700 °C			
				steam = 5 mL h^–1^			
10Ni-Ca-C	PS-pine sawdust (5:5)	cp	fixed/fixed (0.5 g mixture)	*T*_P_ = 800 °C	38.5	13.2	Chai et al.^166^
				*T*_R_ = 700 °C			
				steam = 5 mL h^–1^			

a cp, Co-impregnation.

b *T*_P_ = Pyrolysis temperature; *T*_R_ = reforming temperature.

More recently, a new dual-support catalyst Ni-CaO-C was used by Chai et al.^166^ with the aim of enhancing the H_2_ production in the catalytic reforming of the volatiles from the pyrolysis of different plastics (HDPE, PP, and PS) and biomass (pine sawdust) using a feedstock with a biomass/plastic ratio of 5:5. The results revealed that H_2_ production decreased depending on the type of plastic mixed with the biomass, as follows: HDPE (14.0 wt %) > PP (13.8 wt %) > PS (13.2 wt %).

**Figure 14:**
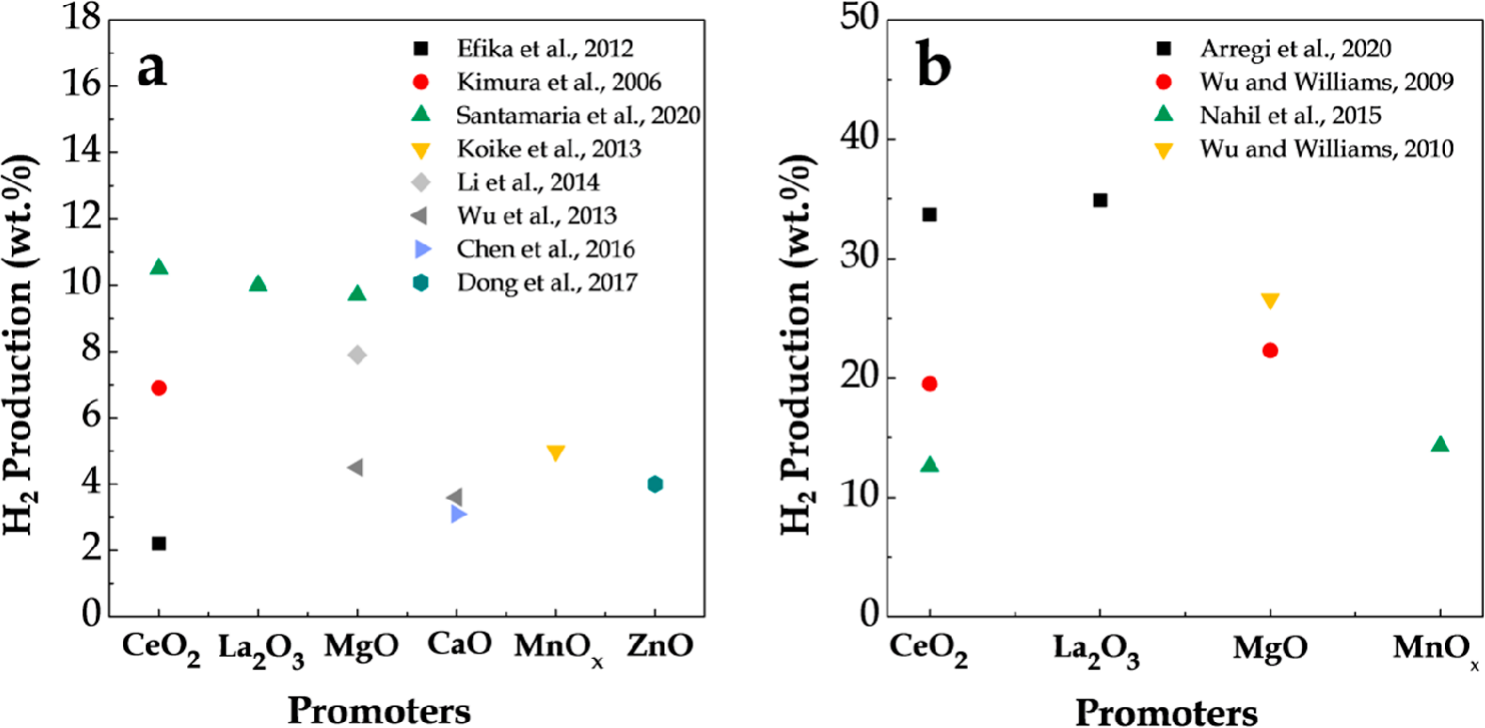
Influence of the promoter in Ni-Al_2_O_3_ catalysts on the H_2_ production in the in-line pyrolysis-reforming process, when biomass (a) and polypropylene (PP) (b) are in the feed. Efika et al., 2012;^102^ Kimura et al., 2006;^124^ Santamaria et al., 2020;^191,192^ Koike et al., 2013;^88^ Li et al., 2014;^87^ Wu et al., 2013;^195^ Chen et al., 2016;^82^ Dong et al., 2017;^196^ Arregi et al., 2020;^55^ Wu and Williams, 2009;^164^ Nahil et al., 2015;^197^ Wu and Williams, 2010.^47^

A comparison of the main Ni/Al_2_O_3_ promoters used in the literature for the two-step pyrolysis-reforming strategy is shown in Figure 14. Thus, Figure 14a summarizes the studies dealing with biomass used as raw material, whereas Figure 14b displays the ones concerning to the use of polypropylene (PP) as feedstock (the use of other different types of plastics has been scarcely studied, and therefore the influence the promoter has on the reforming of other plastic wastes pyrolysis volatiles cannot be assessed).

As observed in Figure 14, CeO_2_, La_2_O_3_, and MgO are the most widely used promoters in the literature for the pyrolysis-reforming of biomass and plastic wastes. It is to note that, although the incorporation of a promoter can positively contribute to improving catalyst activity, the design of promoted catalysts is usually focused on enhancing catalyst stability by attenuating the fast deactivation caused by coke deposition. In this regard, the results provided in Figure 14 only give an idea of the initial catalyst activity, and therefore the stability of these catalysts must also be considered in further studies.

Moreover, the use of rare earth oxides, such as La_2_O_3_ and CeO_2_ as promoters, has led to encouraging results in the continuous steam reforming of the pyrolysis volatiles derived from biomass^191,192^ and plastic wastes (PP).^55^ Thus, the addition of CeO_2_ as promoter improves considerably the Ni/Al_2_O_3_ catalyst stability, which is related to the CeO_2_ redox properties that increase the surface available for oxygen as well as to its higher water adsorption capacity that enhances coke precursor gasification and attenuates catalyst deactivation. The incorporation of La_2_O_3_ reduces the acidity of the Al_2_O_3_ support, so coke formation and its water adsorption capability promotes the gasification of coke deposits. Thus, under suitable operating conditions, catalyst stability is improved and H_2_ productions of around 10.5 wt % and 35 wt % are obtained when biomass and PP are used as the feedstock, respectively.^55,191,192^

The use of alkaline-earth metals (Mg and Ca) and transition metal oxides (MnO_*x*_, ZnO) as promoters has also been analyzed in the literature, since these basic materials can reduce catalyst acidity and so coke formation.^196^ However, the results shown in Figure 14 are also influenced by the following factors: (i) synthesis conditions (preparation method, calcination temperature, metal loading); (ii) reactor configuration; (iii) the operating variables in the pyrolysis (which greatly conditions the composition of the volatile stream to be reformed) and reforming steps; and (iv) process operation (batch or continuous mode). Thus, a more detailed research must be conducted to evaluate catalysts stability and regenerability and therefore step further toward the scalability of this two-step pyrolysis-reforming process for the production of H_2_ from biomass and plastic wastes.

### 4.4 Bimetallic and Non-Nickel Based Catalysts

The catalytic performance highly depends on the active phase. As shown before, Ni is the most widely used active phase in the literature due to its high activity in the reforming reactions and moderate cost. However, other metals have been evaluated for the pyrolysis and in-line steam reforming of biomass, plastic wastes, and their mixtures. Among these metals, transition metals, such as Fe, Co, or Cu, and noble metals, such as Rh, Pt, Pd, and Ru are worth mentioning. Moreover, these metals have also been added as secondary metals forming bimetallic catalysts, which may improve catalytic activity and coke resistance. Thus, Tables 13 and 14 summarize the studies reported in the literature for non-nickel based catalysts and bimetallic catalysts, respectively, for the biomass derived feedstock, and Table 15 shows the ones when plastic wastes are used as feedstock.

**Table 13:** Non-Ni Based Catalysts Reported in the Literature for Biomass Pyrolysis and In-line Steam Reforming

Catalyst	Feed	Preparation method^b^	Reactor configuration	Operating conditions^d^	H_2_ conc. (vol %)	H_2_ prod. (wt %)	Ref.
10Co/Al_2_O_3_	pinewood sawdust	wi	spouted/fluidized (0.75 g min^–1^)	*T*_P_ = 500 °C	39.1	2.3	Santamaria et al.^199^
				*T*_R_ = 600 °C			
				S/C = 7.7			
12Co/Al_2_O_3_	cedar wood	iwi	dual fixed (0.06 g min^–1^)	*T* = 550–650 °C	58.1	8.0	Li et al.^86^
				S/C = 0.47			
12Co/ZrO_2_	cedar wood	iwi	dual fixed (0.06 g min^–1^)	*T* = 550–650 °C	49.0	5.3	Li et al.^86^
				S/C = 0.47			
12Co/SiO_2_	cedar wood	iwi	dual fixed (0.06 g min^–1^)	*T* = 550–650 °C	43.3	4.2	Li et al.^86^
				S/C = 0.47			
12Co/MgO	cedar wood	iwi	dual fixed (0.06 g min^–1^)	*T* = 550–650 °C	44.4	4.6	Li et al.^86^
				S/C = 0.47			
12Co/TiO_2_	cedar wood	iwi	dual fixed (0.06 g min^–1^)	*T* = 550–650 °C	23.9	1.6	Li et al.^86^
				S/C = 0.47			
CaO/MgO	sugar cane leaves	wm	fixed/fixed (0.12 g)	*T*_P_ = 400–800 °C		4.3	Bunma and Kuchonthara^200^
				*T*_R_ = 600–800 °C			
12Co/BaAl_12_O_19_	cedar wood	iwi	dual fixed (0.06 g min^–1^)	*T* = 550–650 °C	59.1	8.5	Li et al.^86^
				S/C = 0.47			
Rh/CeO_2_-SiO_2_	cedar wood	iwi	fluidized/fluidized (0.15 g min^–1^)	*T* = 650 °C	44.6	4.7	Tomishige et al.^96^
Pt/CeO_2_-SiO_2_	cedar wood	iwi	fluidized/fluidized (0.15 g min^–1^)	*T* = 650 °C	42.2	4.2	Tomishige et al.^96^
Pd/CeO_2_-SiO_2_	cedar wood	iwi	fluidized/fluidized (0.15 g min^–1^)	*T* = 650 °C	40.5	3.8	Tomishige et al.^96^
Ru/CeO_2_-SiO_2_	cedar wood	iwi	fluidized/fluidized (0.15 g min^–1^)	*T* = 650 °C	18.9	1.0	Tomishige et al.^96^
2.5Fe/CS^a^	apple branch	wi	fixed/fixed (0.6 g)	*T* = 650 °C	50.3	3.7^c^	Guan et al.^183^
0.5Fe/zeolite	seaweed	iwi	fixed/fixed (0.6 g)	*T* = 510–660 °C	48.6	4.1	Kaewpanha et al.^184^
Fe/ZnO-Al_2_O_3_	wood sawdust	cp	fixed/fixed (0.5 g)	*T*_P_ = 500 °C	41.0	1.9	Chen et al.^81^
				*T*_R_ = 800 °C			
0.5Rh/zeolite	seaweed	iwi	fixed/fixed (0.6 g)	*T* = 510–660 °C	52.8	5.3	Kaewpanha et al.^184^

a CS, calcined scallop shell.

b iwi, Incipient wetness impregnation; wi, wet impregnation; wm, wet mixing; cp, co-precipitation.

c H_2_ production defined as g H_2_/100 g biomass, daf (dry and ash free).

d *T*_P_ = Pyrolysis temperature; *T*_R_ = reforming temperature.

**Table 14:** Bimetallic Catalysts Reported in the Literature for Biomass Pyrolysis and In-line Steam Reforming

Catalyst	Feed	Preparation method^b^	Reactor configuration	Operating conditions^c^	H_2_ conc. (vol %)	H_2_ prod. (wt %)	Ref.
5Ni5Co/Al_2_O_3_	pinewood sawdust	wi	spouted/fluidized (0.75 g min^–1^)	*T*_P_ = 500 °C	63.4	9.8	Santamaria et al.^199^
				*T*_R_ = 600 °C			
				S/C = 7.7			
7.5Ni2.5Co/Al_2_O_3_	pinewood sawdust	wi	spouted/fluidized (0.75 g min^–1^)	*T*_P_ = 500 °C	63.6	9.9	Santamaria et al.^199^
				*T*_R_ = 600 °C			
				S/C = 7.7			
Ni-Fe/Al_2_O_3_	cedar wood	cp	dual fixed (0.06 g min^–1^)	*T* = 550 °C	50.6	5.2	Wang et al.^201^
				S/C = 0.57			
Co-Fe/Al_2_O_3_	cedar wood	cp	dual fixed (0.06 g min^–1^)	*T* = 600 °C	50.3	6.0	Wang et al.^202^
				S/C = 0.57			
Ni-Cu/MgO-Al_2_O_3_	cedar wood	cp	dual fixed (0.06 g min^–1^)	*T* = 650 °C	55.2	8.3	Li et al.^87^
				S/C = 0.38			
0.1Pt-4Ni/CeO_2_-Al_2_O_3_	cedar wood	wi	dual fixed (0.06 g min^–1^)	*T* = 550 °C	54.1	6.1	Nishikawa et al.^203^
0.1Rh-4Ni/CeO_2_-Al_2_O_3_	cedar wood	wi	dual fixed (0.06 g min^–1^)	*T* = 550 °C	48.0	4.6	Nishikawa et al.^203^
0.5Ru-4Ni/CeO_2_-Al_2_O_3_	cedar wood	wi	dual fixed (0.06 g min^–1^)	*T* = 550 °C	47.4	4.5	Nishikawa et al.^203^
Ni-Fe/RHA^a^	rice husk	iwi	fixed/fixed	*T*_P_ = 800 °C	31.5	5.5	Shen et al.^185^
				*T*_R_ = 600–900 °C			
Ni-Fe/RHC^a^	rice husk	iwi	fixed/fixed	*T*_P_ = 800 °C	22.7	4.3	Shen et al.^185^
				*T*_R_ = 600–900 °C			

a RHA, Rice husk ash; RHC, rice husk char.

b iwi, Incipient wetness impregnation; wi, wet impregnation; cp, co-precipitation.

c *T*_P_ = Pyrolysis temperature; *T*_R_ = reforming temperature.

**Table 15:** Bimetallic and Non-Ni Based Catalysts Reported in the Literature for the Pyrolysis and In-line Steam Reforming of Plastic Wastes

Catalyst	Feed	Preparation method^b^	Reactor configuration	Operating conditions^c^	H_2_ conc. (vol %)	H_2_ prod. (wt %)	Ref.
4.4Ru/Al_2_O_3_	PS	commercial (AP4002)	fixed/fixed (1 g min^–1^)	*T*_P_ = 400–600 °C	68.2	33.0	Namioka et al.^90^
				*T*_R_ = 580–680 °C			
				S/C = 3.7 (molar)			
0.5Ru/Al_2_O_3_	PP	commercial (AP4002)	fixed/fixed (1 g min^–1^)	*T*_P_ = 400–550 °C	54.0	4.5	Park et al.^43^
				*T*_R_= 630 °C			
				S/C = 3.6 (molar)			
5Ru/Al_2_O_3_	PP	commercial (AP4002)	fixed/fixed (1 g min^–1^)	*T*_P_= 400–600 °C	69.8	36.5	Park et al.^43^
				*T*_R_= 580–680 °C			
				S/C = 3.7 (molar)			
20Fe/ZrO_2_	PS	cp	fixed/fixed (300 mg)	*T*_P_ = 500 °C	81.5	2.6	Zhou et al.^172^
				*T*_R_= 500 °C			
				steam = 0.02 mL min^–1^			
Fe-Ni-MCM-41	SMWPs^a^	wi	fixed/fixed	*T*_P_ = 500 °C	46.7	9.2	Zhang et al.^204^
				*T*_R_ = 800 °C			
				steam = 2 mL h^–1^			
5Ni-15Fe/ZrO_2_	PS	cp	fixed/fixed (0.3 g)	*T*_P_ = 500 °C	69.5	6.9	Zhou et al.^172^
				*T*_R_ = 500 °C			
				steam = 0.02 mL min^–1^			
10Ni-10Fe/ZrO_2_	PS	cp	fixed/fixed (0.3 g)	*T*_P_ = 500 °C	69.0	7.4	Zhou et al.^172^
				*T*_R_ = 500 °C			
				steam = 0.02 mL min^–1^			
15Ni-5Fe/ZrO_2_	PS	cp	fixed/fixed (0.3 g)	*T*_P_ = 500 °C	63.0	8.6	Zhou et al.^172^
				*T*_R_ = 500 °C			
				steam = 0.02 mL min^–1^			
NiCuAl 1:1:2	PP	cp	fixed/fixed (1 g)	*T*_P_ = 500 °C	61.1	18.9	Wu and Williams^150^
				*T*_R_ = 800 °C			
				steam = 4.74 g h^–1^			
NiCuMgAl	PP	cp	fixed/fixed (1 g)	*T*_P_ = 500 °C	62.2	20.4	Wu and Williams^150^
				*T*_R_ = 800 °C			
				steam = 4.74 g h^–1^			

a SMWPs, Simulated mixed waste plastics. (LDPE, 42 wt %; HDPE, 20 wt %; PS, 16 wt %; PET, 12 wt % PP, 10 wt %).

b iwi, Incipient wetness impregnation; wi, wet impregnation; cp, co-precipitation.

c *T*_P_ = Pyrolysis temperature; *T*_R_ = reforming temperature.

Consequently, Li et al.^86^ studied different Co based catalysts supported on Al_2_O_3_, ZrO_2_, SiO_2_, MgO, TiO_2_, and BaAl_12_O_19_ (BA) in the steam reforming of the tar from the pyrolysis of wood biomass. The highest catalytic activity was obtained when Co/BA was used, which was attributed to the high dispersion obtained on this strongly basic support. Moreover, the highest H_2_ production was obtained on Co/BA catalyst (8.5 wt %), followed by Co/Al_2_O_3_ catalyst (8.0 wt %).

Kaewpanha et al.^184^ tested different metal catalysts, i.e., Ni, Fe, and Rh supported on commercial zeolite, in the steam reforming of the tar derived from the steam pyrolysis of seaweed in a fixed bed reactor. They found that the highest H_2_ production was obtained at a reaction temperature above 610 °C, with Rh/zeolite catalyst being the most effective for tar removal, with a H_2_ production of 5.3 wt %. Tomishige et al.^96^ investigated the steam reforming of biomass-derived tars on several noble metal based catalysts (Rh, Pt, Pd, and Ru) supported on CeO_2_-SiO_2_. The experiments were conducted in a laboratory scale continuous feeding device, which was made up of a primary bed for pyrolysis of biomass and a secondary fluidized catalytic bed for the reforming step. They reported that the activity order at 550 °C was as follows: Rh > Pt > Pd > Ru.

Moreover, although noble metal catalysts enhance catalyst activity and reduce coke deposition, their high cost limits their use as a single active phase. Therefore, their joint used with cheaper metals, such as Ni, in order to form bimetallic catalysts, is an interesting option followed by many authors. Similarly, with the aim of improving the overall activity of the catalysts, the addition of various transition metals to form different alloys has been approached in the literature.

The performance of Ni-Fe/Al_2_O_3_ catalysts in the steam reforming of the tar from the pyrolysis of cedar wood was analyzed by Wang et al.^201^ obtaining higher activity than that corresponding to monometallic Ni and Fe catalysts. The alloy formed between Ni and Fe improved the reaction involving the tar and hindered coke formation, since oxygen atoms are supplied by Fe species. Similarly, this research group analyzed the performance of Fe-Co/Al_2_O_3_, reporting its higher activity and stability compared to Fe/Al_2_O_3_ and Co/Al_2_O_3_ catalysts. Thus, the H_2_ production obtained with the bimetallic catalyst was 6.0 wt %.^202^

Moreover, Santamaria et al.^199^ studied the effect the active phase has on the performance of the reforming catalysts. The metals selected as active phase were Ni, Co, and two bimetallic Ni-Co catalysts with different loading ratios. The runs were conducted in a continuous lab scale unit provided with a conical spouted bed reactor for the pyrolysis step and a fluidized bed reactor for the reforming of the volatiles formed in the first step. The H_2_ production on these catalysts decreased as follows: Ni/Al_2_O_3_ (10.17 wt %) > 7.5Ni-2.5Co/Al_2_O_3_ (9.94 wt %) > 5Ni-5Co/Al_2_O_3_ (9.82 wt %) ≫ Co/Al_2_O_3_ (2.3 wt %). Moreover, the poor initial performance of Co/Al_2_O_3_ catalyst at zero time on stream was attributed to the oxidizing nature of the steam, which favored the conversion of Co^0^ into inactive CoO phase.

In addition, the influence of incorporating noble metals into Ni based catalysts was investigated by Nishikawa et al.^203^ Thus, Pt, Pd, Rh, and Ru were incorporated into a Ni/CeO_2_-Al_2_O_3_ catalyst, and Pt was the most effective catalyst, even when its loading amount was low (0.01 wt %), which is due to the strong interaction between Pt and Ni to form the alloy. Moreover, the activity order attained was as follows: Pt > Rh > Ru > Pd.

**Figure 15:**
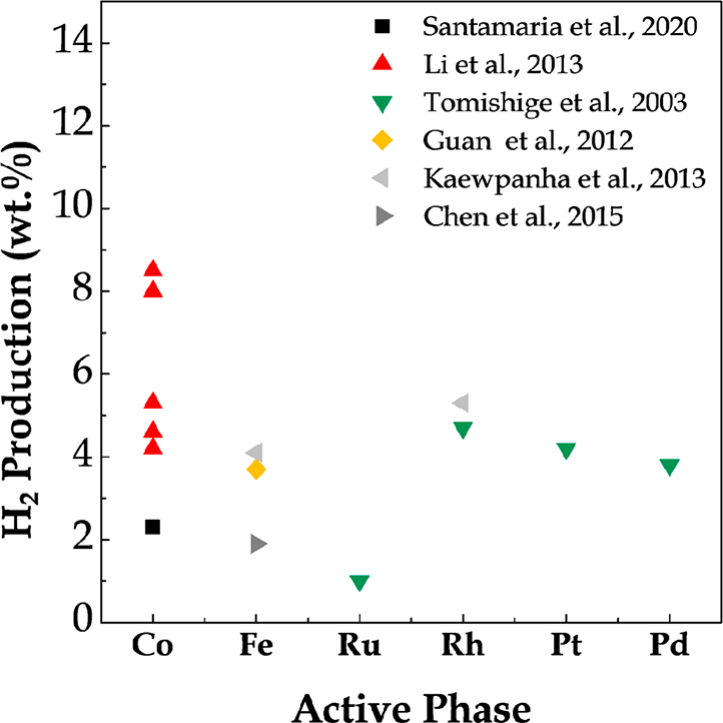
Influence of the active phase on H_2_ production in the in-line process of biomass pyrolysis and steam reforming. Santamaria et al., 2020;^199^ Li et al., 2013;^86^ Tomishige et al., 2003;^96^ Guan et al., 2012;^183^ Kaewpanha et al., 2013;^184^ Chen et al., 2015.^81^

The H_2_ productions reported in the literature using different active phases in the biomass pyrolysis and in-line steam reforming are summarized in Figure 15. As previously mentioned, most of these studies have been conducted using transition metals, such as Fe and Co, due to their lower cost compared to noble metals. As observed, a maximum H_2_ production of 8.5 wt % was obtained in a dual fixed bed reactor, using Co/BaAl_12_O_19_ as reforming catalyst.^86^ The lower H_2_ productions obtained with metal phases alternative to Ni active phase have led to the use of bimetallic catalysts. However, it should be noted that the results provided in Figure 15 are greatly conditioned by the type of the support used (apart from the reaction conditions). Therefore, any comparison of the results obtained in the literature involves great difficulties. In fact, few studies deal with the influence the metal active phase and bimetallic catalysts have on H_2_ production in the two-step pyrolysis-reforming process.

Similarly, the use of bimetallic and non-nickel based catalysts has also been assessed in the pyrolysis and in-line steam reforming of plastic wastes. Thus, Park et al.^43^ conducted a parametric study of the two step pyrolysis-reforming of PP using two fixed bed reactors operating in the continuous regime (1 g min^–1^) and two commercial Ru/Al_2_O_3_ catalysts with different Ru loadings (0.5 and 5 wt %). The highest H_2_ production (36.5 wt %) was reported when the 5Ru/Al_2_O_3_ catalyst was used under the following operating conditions: pyrolysis temperature of 400 °C, reforming temperature of 680 °C, and S/C ratio of 3.7. The use of PS as feedstock was analyzed by the same authors in the same experimental unit at pyrolysis and reforming temperatures of 400 and 630 °C, respectively.^90^ Under these conditions, they reported a slightly lower H_2_ production when PS was fed (33.0 wt %) compared to the previous work, wherein they obtained a H_2_ production of 34.2 wt % using PP as the feedstock.

Recently, the production of high H_2_/CO gas in the steam reforming of PS volatiles was analyzed by Zhou et al.^172^ in a fixed bed reactor provided with two-zone horizontal furnaces. Ni-Fe bimetallic catalysts supported on ZrO_2_ were synthesized by a co-impregnation method. The results showed that, although Fe/ZrO_2_ was not an effective catalyst for this process, the bimetallic Ni-Fe/ZrO_2_ catalyst could catalyze the reaction efficiently and produce a much higher H_2_ compared to the monometallic catalyst (8.6 wt % for 15Ni5Fe/ZrO_2_ and 2.6 wt % for 20Fe/ZrO_2_ catalyst).

## 5 Challenges and Perspectives

In recent decades, the dependency of fossil fuels for energy production has triggered an increasing concern for the environmental problems associated with CO_2_ emissions and global warming. Within this scenario, H_2_ is regarded as one of the future energy carriers, and the development of new sustainable routes for H_2_ production from biomass and plastic wastes is therefore a pressing need for the market. Among the different thermochemical routes for H_2_ production, the two-step pyrolysis and in-line steam reforming strategy has gained increasing attention. Thus, this strategy provides several advantages compared to the conventional steam gasification and steam reforming of the bio-oil or plastic pyrolysis oil, as are: (i) operation under optimum conditions due to the integration of the two reactors in the same unit (the reforming temperature is lower than the one used in gasification process, thus reducing possible sintering problems of the catalyst); (ii) avoidance of tar formation; (iii) direct contact between the feedstock and its impurities with the reforming catalyst is avoided; and (iv) higher H_2_ productions are attained (above 10 and 30 wt % when biomass and plastic wastes are used, respectively). Besides, the versatility of this process allows co-feeding biomass and plastic mixtures, reducing the limitations derived from seasonal biomass, and decreasing the environmental problems related to the management of plastic wastes.

Although great effort has been made to analyze in detail the optimum operating conditions in both steps, (i.e., pyrolysis and reforming temperatures, S/B or S/P ratio, space time, and so on), most of these studies have been conducted in laboratory reactors, which operate in batch mode. In fact, the studies performed in the continuos regime are restricted to bench scale units. Thus, the degree of development of pyrolysis-reforming technologies must be improved in order to ensure their scale up and implementation. Accordingly, special attention should be paid to the reactor design. Fluidized and spouted beds are those with better perspectives for the pyrolysis step. Thus, fast pyrolysis conditions allow obtaining high selectivity toward volatile compounds, especially bio-oil and plastic derived oil, with low solid byproduct formation. Accordingly, H_2_ production in the overall pyrolysis-refoming process can be enhanced. Regarding the selection of the reforming reactor, two configurations have been analyzed in the previous literature, fixed and fluidized beds. Fixed beds are of easy design and operation, with catalyst attrition problems being avoided. However, fluidized beds have significant operational advantages over fixed beds for the full-scale operation. Thus, the solid circulation in fluidized beds ensures a better temperature control and avoids temperature gradients in the catalytic bed, which involves a remarkable challenge in highly endothermic steam reforming reactions. Moreover, the reforming of biomass and plastics derived pyrolysis volatiles faces a fast deactivation rate, mainly associated with coke deposition. In this respect, a suitable reaction-regeneration strategy must be considered for the process scale up. Fluidized bed reactors are more flexible than fixed beds, as reaction-regeneration strategies with continuous catalyst circulation between the reforming reactor and catalyst regenerator can be developed. Furthermore, the development of this process is greatly conditioned by the catalyst design, in which the catalytic materials and synthesis method and conditions are crucial facts. Accordingly, the main challenges to overcome to step further in the scaling-up of this two-step process are as follows: (i) the implementation of a continuous process in any reactor configuration, andconfiguration; and, (ii) the fast catalyst deactivation in the reforming step. It is worthy to note that, although the scaling-up of this strategy has not been implemented yet, certain research studies have proposed a continuous pyrolysis-reforming operation in bench scale units.

Future research is needed to acquire further knowledge on reforming catalysts in order to progress toward the industrial scale. Special attention should be paid to the studies dealing with the mechanisms of catalyst deactivation and the role played by the catalyst design in the attenuation of catalyst deactivation. Thus, the influence catalytic materials, the synthesis method, and conditions have on the catalyst stability in the reforming step must be addressed. Alternative strategies may also be proposed for improving the fast catalyst deactivation, as are those involving the modification of the volatile stream to be reformed, which may be conducted in the pyrolysis reactor itself or downstream the pyrolysis reactor.

Besides, studies dealing with the regenerability of the reforming catalysts are essential for a suitable scaling of the pyrolysis-reforming process, since large scale plants entail working under reaction-regeneration cycles. Thus, prior to proceed with the optimum discrimination of catalysts, regeneration studies should be conducted.

## 6 Conclusions

This review analyzes the background and state-of-the-art of metal catalysts for the steam reforming of the volatile stream derived from the pyrolysis of biomass and waste plastics. In recent years, increasing research studies have focused on this strategy, since it has been demonstrated to be a suitable process for direct H_2_ production.

The development of suitable reforming catalysts is essential for the viability of this process. Thus, a wide range of materials has been analyzed in the literature for improving the catalyst activity and stability in H_2_ production by pyrolysis-reforming of biomass and plastic wastes. It should be noted that non-metal based catalysts have not been included in this review due to their lower activity in the reforming reaction. The Ni/Al_2_O_3_ catalyst has been extensively used due to its moderate cost, high activity of Ni metal, and suitable properties conferred by the Al_2_O_3_ support (high specific surface area, mechanical strength, and stability) upon the catalyst. However, the acid nature of the Al_2_O_3_ support, which promotes coke formation on the catalyst, has led to the substitution of Al_2_O_3_ by other supports, both metal oxides, such as MgO, ZrO_2_, TiO_2_ or CeO_2_, and low-cost materials, such as biomass and coal derived char, active carbon, dolomite, and zeolites. Among them, ZrO_2_ is regarded as a promising catalyst support for its use in reaction-regeneration cycles. Conversely, the problems associated with the regeneration of the char and active carbon supported catalysts may condition its use in large scale units.

Incorporation of promoters has also been assessed in order to enhance catalyst stability in the steam reforming of the volatiles derived from the pyrolysis of biomass and plastic wastes. Thus, La_2_O_3_ and CeO_2_ have led to promising results for the attenuation of catalyst deactivation. The features of these promoters, i.e., basicity and water adsorption capability of La_2_O_3_ and redox properties of CeO_2_ promoter, involve a reduction in the formation of coke deposits and therefore improve the stability of Ni/Al_2_O_3_ catalysts. Besides, the basic properties of other metal oxides, such as MgO, CaO, and MnO_*x*_ have boosted their use as catalytic promoters.

Similarly, transition metals (mainly Co and Fe), noble metals (Rh, Ru, Pt, and Pd) and bimetallic catalysts have been tested in this two-step strategy, although these studies are still scarce.

A comparison of the diverse results reported in the literature involves great difficulty. Thus, apart from the catalyst selected in the reforming step, the overall H_2_ production in the pyrolysis-reforming process from biomass, plastic wastes, and their mixtures greatly depends on the following factors: (i) type of feedstock, which must consider biomass/plastic features, particle size, moisture content, and ash composition; (ii) feeding regime, with continuous feed allowing for reaching the steady state and controlled operating conditions; (iii) pyrolysis step, in which the type of reactor, heating rate, gas residence time, and temperature greatly condition the volatile stream to be reformed; (iv) reforming conditions, as are type of reactor, S/B or S/P ratio, space time, and temperature; and (v) catalyst design, i.e., preparation method, catalytic materials, and synthesis conditions (calcination temperature, metal loading, reduction temperature).

Although encouraging results have been obtained based on this strategy, further knowledge of reforming catalysts is required in order to progress toward the industrial scale. Thus, special attention should be paid to the studies dealing with the mechanisms of catalyst deactivation and regeneration in order to proceed with the optimum discrimination of catalysts.
